# Efficacy of micro-video psychological training camp for reducing depression and anxiety and enhancing resilience: a randomized controlled trial

**DOI:** 10.1186/s12888-026-07807-6

**Published:** 2026-01-23

**Authors:** Wenqing Zhao, Wanlin Tang, Shuangyi Chen, Jiayu Yao, Jun Hu, Qing Zhou, Jing Tao, Rui Gao, Jie Zhang, Yanru Wu, Shanshan Su, Yuan Wang, Yousong Su, Yihua Peng, Yating Zhao, Qing Fan, Weibo Zhang, Wenhui Jiang, Jun Cai, Jianyin Qiu

**Affiliations:** 1 Shanghai Mental Health Center, Shanghai Jiao Tong University School of Medicine, Shanghai, China; 2 Shanghai Xuhui Mental Health Center, Shanghai, China

**Keywords:** Depression and anxiety symptoms, Psychological resilience, Self-service, Psychological intervention, Randomized controlled trial (RCT)

## Abstract

**Background:**

Depression and anxiety disorders are highly prevalent and frequently co-occur, a comorbidity associated with greater chronicity and symptom severity. Access to evidence-based psychological interventions remains limited, particularly in resource-constrained settings. Digital interventions offer a scalable solution. This study evaluated the feasibility, acceptability, and preliminary efficacy of the Micro-Video Psychological Training Camp (MVPTC), a fully automated digital intervention based on integrative psychotherapy principles and targeting mild-to-moderate depression/anxiety and multidimensional mental health.

**Methods:**

In this randomized controlled trial, 204 adults (aged 18–70) with mild-to-moderate symptoms were allocated to either the MVPTC intervention (8 self-guided sessions, *n* = 97) or waitlist control group (*n* = 107). The severity of depressive and anxiety symptoms served as primary outcomes, and psychological resilience as a secondary outcome. Exploratory outcomes included sleep quality, coping strategies, and perceived social support. All were measured at baseline, post-intervention, and at 1-month and 3-month follow-ups. Feasibility (dropout and adherence rates) and acceptability (satisfaction ratings) were also assessed.

**Results:**

Participants (mean age 30.68 years, SD 7.6; 66.2% female, 33.8% male) exhibited a 36.08% dropout rate in the MVPTC group versus 8.41% in control group. The intervention led to significant, sustained reductions in depressive symptoms (Cohen’s *d* = 0.87 at T2, 0.99 at T3, 0.80 at T4; all *P* < 0.01) and anxiety symptoms (Cohen’s *d* = 0.71 at T2, 0.75 at T3, 0.63 at T4; *P* ≤ 0.01). Significant improvements were also observed in psychological resilience (Cohen’s *d* =-0.97 at T3, -0.84 at T4; *P* < 0.01) and sleep quality (ISI: Cohen’s *d* = 0.97–1.08; *P* ≤ 0.01). Coping strategies improved transiently (Cohen’s *d* =-0.58 at T2, -0.53 at T3; *P* < 0.05), while perceived social support showed no sustained group differences despite temporary family/friend support increases at T3 (Cohen’s *d* =-0.55 to -0.68; *P* < 0.05). Participants rated the intervention as acceptable, with an average satisfaction score of 3.06 out of 4 (SD = 0.41; *n* = 44).

**Conclusions:**

The MVPTC is a promising, fully automated digital intervention that significantly and sustainably reduced core symptoms of depression and anxiety while enhancing psychological resilience and sleep quality over a 3-month period in adults with mild-to-moderate symptoms. Its effects on coping strategies and social support were less enduring. The program demonstrates potential for scalable implementation. Future research should focus on strategies to improve adherence and evaluate its effectiveness in broader, real-world settings.

**Trial registration:**

Registered on Chinese Clinical Trial Registry (ChiCTR2100043725). Registered: February 27, 2021, http://www.chictr.org.cn/.

**Supplementary Information:**

The online version contains supplementary material available at 10.1186/s12888-026-07807-6.

## Introduction

Depression and anxiety disorders are among the most prevalent mental health conditions worldwide, representing a critical public health challenge with a substantial burden on society and the economy [1, 2]. Although effective treatments such as pharmacotherapy and psychotherapy exist, a substantial treatment gap persists globally, leaving over half of affected individuals lacking adequate care [3, 4]. This gap is particularly pronounced in China, where access is hindered by multiple barriers: pervasive stigma, limited mental health literacy, a severe shortage of trained providers, inadequate service availability, long waiting times and high costs—especially in underserved areas [5–7]. These systemic challenges highlight the urgent need to develop, evaluate, and implement more accessible and scalable forms of evidence-based psychological intervention.

Depression and anxiety disorders frequently co-occur, with epidemiological studies estimating concurrent presentation in approximately 40–60% of cases in the general population, rising to 50–70% in clinical settings [8]. This comorbidity is clinically significant, as it is associated with greater symptom severity, increased chronicity, and a more complex treatment course compared to either condition alone [9, 10]. Contemporary research supports a transdiagnostic perspective, suggesting that such co-occurrence arises from shared underlying psychological mechanism [11, 12]. Key transdiagnostic mechanisms include persistent maladaptive cognitive patterns (e.g., rumination, catastrophizing), emotion dysregulation, behavioral avoidance, and interpersonal dysfunction, which together constitute a “negative affect syndrome” [13–17].

These mechanisms are not merely correlated symptoms but active, interacting processes that maintain comorbidity. Persistent negative cognitive patterns (e.g., rumination) act as an engine for negative affect, selectively processing threat- or loss-related information to fuel both anxiety and depression [18]. Deficits in emotion regulation amplify and prolong distress by promoting maladaptive strategies (e.g., suppression, avoidance), thereby increasing vulnerability to episodes [19]. Behavioral avoidance prevents fear extinction in anxiety and reduces engagement in rewarding activities in depression, negatively reinforcing dysfunctional beliefs [20, 21]. Interpersonal difficulties erode social support, creating a vicious cycle of perceived rejection and heightened stress reactivity [22, 23]. Critically, these mechanisms interact dynamically (e.g., rumination intensifies sadness, prompting withdrawal, which then fuels worthlessness and strains relationships), explaining the complexity and chronicity of comorbid presentations. Consequently, interventions targeting only a single facet may be insufficient.

Transdiagnostic interventions, designed to address these common maintenance factors simultaneously, have demonstrated efficacy comparable to disorder-specific therapies in alleviating symptoms of both depression and anxiety [15, 24]. However, access to traditional face-to-face interventions remains limited. Internet-delivered psychotherapy has thus emerged as a promising approach to bridge this treatment gap [25]. Digital transdiagnostic interventions have been shown to effectively reduce symptoms of anxiety and depression while also improving overall quality of life [26].

While most digital interventions for depression and anxiety are based on Cognitive Behavioral Therapy (CBT), with only a minority incorporating mindfulness [27], there is growing interest in integrative approaches that combine techniques from multiple evidence-based modalities. An integrative framework represents a mechanism-targeted, synergistic strategy [17]. Beyond combining core CBT and mindfulness components, it may incorporate skill modules derived from Dialectical Behavior Therapy (DBT) to enhance emotion regulation and principles from Interpersonal Psychotherapy (IPT) to address interpersonal difficulties [22, 28, 29]. By concurrently targeting these interconnected core mechanisms, an integrated intervention can more comprehensively disrupt the maintaining cycles of comorbid depression and anxiety than a single-modality approach [30].

In the Chinese context, leveraging ubiquitous mobile platforms like WeChat and employing engaging formats such as micro-videos offers a unique opportunity to enhance user engagement and cultural acceptability [31, 32]. Despite this potential, few studies have evaluated the efficacy of a fully automated, integrative, transdiagnostic digital intervention delivered via such platforms for individuals with mild-to-moderate symptoms in China. Moreover, beyond symptom reduction, fostering psychological resilience—the adaptive capacity to recover from adversity—represents a crucial yet often overlooked treatment target that may confer longer-term protection against symptom relapse [33, 34].

This study aimed to investigate the Micro-Video Psychological Training Camp (MVPTC), a fully automated, self-guided digital intervention delivered via a WeChat Mini Program. Grounded in an integrative psychotherapeutic framework, the MVPTC combines components from CBT, DBT/mindfulness, and IPT within a series of brief, animated videos. We examined its effects on reducing symptoms of depression and anxiety and enhancing psychological resilience in a Chinese sample with mild-to-moderate symptoms, compared to a wait-list control group.

## Methods

### Study design and setting

This was a prospective, two-arm, parallel-designed randomized controlled trial (RCT) comparing th*e* Micro-video Psychological Training (MVPTC) intervention group with a waitlist control group. Assessments were conducted at four time points: baseline (T1), post-intervention (T2), 1-month follow-up (T3), and 3-month follow-up (T4). The study protocol has been published previously [35]. The trial received ethical approval from the Ethics Committee of the Shanghai Mental Health Center (SMHC) (Approval Number: 2020-13) and was registered with the Chinese Clinical Trial Registry (ChiCTR2100043725). This study was conducted in accordance with the CONSORT guidelines [36, 37].

Participants were recruited between February 2021 and December 2023 through multiple channel*s*: the Psychological Counseling Clinic of SMHC, community mental health screening programs, and targeted advertisements on the social media platform WeChat. Recruitment and initial eligibility screening were conducted by licensed psychiatrists and certified psychotherapists via 15-minute structured interviews (face-to-face or by telephone). Potentially eligible individuals then underwent standardized psychological assessments administered by trained researchers. Eligible candidates received comprehensive trial information and provided electronic informed consent prior to enrollment.

### Participants and eligibility

Eligible participants were aged 18–70 years. The original upper age limit was set at 55 years, based on conventional considerations of digital literacy. During the recruitment phase, this limit was pragmatically extended to 70 years on a case-by-case basis to include one highly motivated 68-year-old applicant who self-reported proficiency in using smartphone apps for daily tasks (e.g., messaging, browsing). This constituted a minor protocol deviation. The participant enrolled under this adjusted criterion (representing 0.5% of the final sample) successfully completed the entire intervention and all follow-up assessments.

Clinical eligibility required the presence of mild-to-moderate depressive or anxiety symptoms, defined by a Self-Rating Depression Scale (SDS) scores of 53–72 and/or a Self-Rating Anxiety Scale (SAS) scores of 50–69. Exclusion criteria comprised: (1) severe psychopathology (SDS/SAS scores exceeding the defined thresholds, or a current DSM-5 diagnosis other than depression or anxiety disorders, as assessed by the Mini-International Neuropsychiatric Interview [MINI] [38]; (2) acute suicide ideation or behavior (indicated by a score > 1 on item 20 of the SDS or reported suicidal ideation/behavior within the past month); (3) regular use of psychotropic medication (defined as ≥ 4 days per week for one continuous month or longer) o*r* participation in structured psychotherapy for depression/anxiety in the 6 months prior to enrollment. This criterion aimed to assess the intervention’s initial effect in individuals not currently receiving other active treatments.

### Randomization and blinding

Following baseline assessment, eligible participants were randomly allocated to either the intervention or control group in a 1:1 ratio. The allocation sequence was generated using the random number function in SPSS software (version 23.0), with the official study start date serving as the seed for reproducibility. No stratification factors (e.g., by baseline severity or age) was used. To ensure allocation concealment, the sequence was stored in a password-protected electronic file accessible only to the independent statistician.

After a participant completed the T1 assessment online, a research coordinator (who was blinded to the sequence) contacted the independent statistician. The statistician then revealed the next sequentially numbered allocation exclusively to the coordinator, who subsequently informed the participant of their group assignment via the study platform. This procedure ensured concealment until after baseline data collection.

Given the self-guided nature of the digital intervention, participants could not be blinded to their group assignment. However, to minimize bias, the research assistants responsible for data collection and follow-up communications remained blinded to allocation. Furthermore, the statistician performing the primary efficacy analyses was blinded to group codes until the main outcome analysis was complete.

The final analyzed sample showed an allocation imbalance (97 intervention vs. 107 control). This occurred because 10 participants who were randomized to the intervention group withdrew or were lost to contact after randomization but before completing the baseline assessment. Consequently, they were not included in the baseline dataset or subsequent analyses. All analyses followed the intention-to-treat principle for participants with baseline data.

### Intervention

#### Intervention content and theoretical basis

The Micro-Video Psychological Training Camp (MVPTC) is a fully automated, self-guided digital intervention delivered via a WeChat Mini Program (a lightweight, sub-application within the WeChat ecosystem that requires no separate installation). It is grounded in an integrative psychotherapy framework designed to target the transdiagnostic mechanisms underlying depression and anxiety. The program synthesizes core components from: (1) Cognitive Behavioral Therapy (CBT) for cognitive restructuring and behavioral activation [39, 40]. (2) Mindfulness and Dialectical Behavior Therapy (DBT) skills for emotion regulation, distress tolerance, and present-moment awareness [41–43]. (3) Interpersonal Psychotherapy (IPT) principles to enhance social support and communication [44, 45]. This integrative design aims to holistically address emotional, cognitive, behavioral, and interpersonal functioning.

#### Development and structure

All intervention materials (therapeutic scripts, animated videos, and homework exercises) were developed and iteratively refined by a specialist team at the Shanghai Mental Health Center to ensure theoretical fidelity, clinical accuracy, and cultural relevance. Content is delivered through a series of eight sequentially unlocked, animated micro-videos (3–5 min each), narrated by a virtual therapist (see Supplementary Material S1 for a complete module outline).The modules follow a structured progression: psychoeducation on stress and emotions (Module 1), core skill-building in emotion regulation (Modules 2–3), behavioral activation (Module 4), problem-solving (Module 5), interpersonal skills (Module 6), cognitive restructuring (Module 7), and mindfulness integration for maintenance (Module 8).

#### Procedure, adherence, and engagement

Participants in the MVPTC group were instructed to complete one module every three days over approximately four weeks. The total video content duration was 34.1 min. Each module included several structured homework assignments (e.g., a thought record, a behavioral activation plan, or a mindfulness practice log) designed to translate skills into daily practice. To promote adherence, a sequential locking mechanism was implemented: access to the next module was granted only after the participant had both watched the current module’s video and submitted the corresponding homework. Submission triggered automated, standardized feedback within the platform. Participants could re-watch videos, record practice reflections, and access optional supplemental resources (e.g., mindfulness audio).

#### Safety, monitoring, and concurrent care

The MVPTC was designed as a self-help tool for mild-to-moderate symptoms and did not provide real-time therapeutic support. During informed consent, all participants were explicitly instructed that the program was not for crisis care and were provided with a list of local emergency mental health contacts. In line with standard healthcare confidentiality, there was no routine communication with participants’ family members or guardians. Contact with emergency contacts would only have been initiated in the event of a critical risk disclosure, following a predefined safety protocol approved by the ethics committee. Participants consented to this emergency contact procedure as part of the informed consent process.

A researcher-facing backend system was used to monitor aggregated, anonymized engagement metrics (completion status, viewing timestamps, homework submission) solely for feasibility analysis and to send automated reminders. No personalized therapeutic feedback was provided.

#### Medication and concurrent interventions

Participants were required to be free of regular psychotropic medication (defined as ≥ 4 days per week for one continuous month or longer) and structured psychotherapy for depression/anxiety in the 6 months prior to enrollment. This criterion aimed to assess the intervention’s initial efficacy in a treatment-naïve sample.

During the study, participants were not prohibited from seeking treatment, reflecting real-world conditions. At each assessment (T1-T4), all participants reported any newly commenced treatments. Those who began regular treatment (as defined above) were classified as “treatment changers”. While their data continued to be collected, they were excluded from the primary per-protocol efficacy analysis to conservatively attribute observed effects to the study intervention.

#### Waitlist control group

Participants randomized to the waitlist control group received no active psychological intervention during the study. All assessments were completed online by participants at the four scheduled time points (T1, T2, T3, T4). If a participant did not complete an assessment on time, an automated, standardized reminder was sent via the online platform. No other reminders, psychoeducational content, or therapeutic contact was provided. A predefined safety protocol was established for risk management. If a participant’s questionnaire responses triggered an automated risk alert (e.g., indicating significant suicidal ideation), a research assistant who was blinded to group allocation would initiate the protocol, which included telephone contact for risk assessment and referral to emergency services if necessary. Following completion of the final (T4) assessment, control group participants were granted full access to the MVPTC intervention.

#### Measures

Outcomes were assessed via self-report questionnaires on a secure online platform at baseline (T1), post-intervention (T2), 1-month (T3), and 3-month (T4) follow-ups. Research staff involvement was limited to sending automated reminders and providing technical support.

### Primary outcome

#### Depressive and anxiety symptoms

The severity of depressive and anxiety symptoms was assessed using the Chinese versions of the Self-Rating Depression Scale (SDS) [46] and the Self-Rating Anxiety Scale (SAS) [47], respectively. Both are 20-item self-report measures rated on a 4-point Likert scale, with well-validated Chinese versions demonstrating good reliability (e.g., SDS Cronbach’s α = 0.86; SAS Cronbach’s α = 0.822) [48]. The internal consistency (Cronbach’s alpha) was assessed for both scales in our study. For the SDS, reliability was acceptable at baseline (α = 0.613) and increased to good levels at follow-ups (T2 = 0.820, T3 = 0.862, T4 = 0.871). Similarly, for the SAS, reliability was good at baseline (α = − 0.718) and remained good or improved thereafter (T2 = 0.787, T3 = 0.828, T4 = 0.877). The SDS assesses the frequency and severity of depressed symptoms such as depressed mood, feeling of guilt, loss of interest, appetite, and sleep disturbances. The SAS assesses the frequency and severity of symptoms of subjective feelings of anxiety, such as worry, restlessness, indigestion and difficulty concentrating. Higher scores indicate more severe symptoms. Clinical cut-offs for the SDS are: ≤53 (normal), 53–62 (mild), 63–72 (moderate), ≥ 73 (severe). For the SAS, cut-offs are: ≤50 (normal), 50–59 (mild), 60–69 (moderate), ≥ 70 (severe).

### Secondary outcomes

#### Mental resilience

The Connor-Davidson Resilience Scale (CD-RISC) [49] measures participants’ individual mental resilience levels, which person’s ability and mental resilience to cope with difficulties and adversity. The 25-item Chinese version of the Connor-Davidson Resilience Scale (CD-RISC) [50] comprises three dimensions: tenacity(e.g. “I won’t give up easily when things seem hopeless.”), strength(e.g. “No matter what the result is, I will make every effort to try.”), and optimism(e.g. “I always see the humorous side of things.”) a 5-point Likert scale (1 = almost never to 5 = almost always). It has demonstrated good internal consistency, with a Cronbach’s α of 0.91 for the total scale and 0.88, 0.80, and 0.60 for the respective subscales [51] Higher total scores indicate greater resilience. In the current study, the CD-RISC demonstrated excellent internal consistency across all time points (α = 0.920 at T1, 0.945 at T2, 0.961 at T3, 0.958 at T4).

### Other outcomes

#### Perceived social support

The Perceived Social Support Scale (PSSS), initially developed by Zimet et al. [52] and subsequently adapted and refined by Jiang [53], is a multidimensional tool used to measure the social support perceived by individuals. The scale, which contains 12 items across three subscales (family, friends, significant others), demonstrated good internal consistency in the Chinese sample. Cronbach’s α was 0.88 for the total scale, with subscale α coefficients of 0.87 (Family Support), 0.85 (Friend Support), and 0.91 (Other Support) [54]. In the current study, its internal consistency was excellent across all assessment waves (α = 0.906 at T1, 0.932 at T2, 0.946 at T3, 0.940 at T4). All items are rated on a 7-point Likert scale, with higher scores indicating greater perceived social support.

#### Sleep

The Insomnia Severity Index (ISI) [55] was used to assess sleep disturbances. It consists of 7 items rated 0–4. Total scores categorize sleep as: 0–7 (no clinically significant insomnia), 8–14 (subthreshold insomnia), 15–21 (moderate clinical insomnia), 22–28 (severe clinical insomnia). The Chinese Insomnia Severity Index has shown good psychometric properties (e.g., Cronbach’s α = 0.81) among Chinese community-dwelling older people [56, 57]. In the current study, its internal consistency was excellent and stable across all time points (α = 0.852 at T1, 0.891 at T2, 0.890 at T3, 0.886 at T4).

#### Coping style

The Simplified Coping Style Questionnaire (SCSQ) is a psychological assessment tool designed to evaluate an individual’s coping strategies in response to stress and adversity. Developed by Xie in 1998 [58], the SCSQ is based on the Problem-focused and Emotion-focused model by Lazarus & Folkman (1984) [59]. It consists of 20 items, measuring both positive and negative coping styles, with respondents rating how often they use each strategy on a four-point scale. Previous validation in Chinese samples reported good internal consistency (total α = 0.90; Positive subscale α = 0.89; Negative subscale α = 0.78) [54]. In the current study, the SCSQ demonstrated excellent internal consistency across all waves (α = 0.870 at T1, 0.917 at T2, 0.853 at T3, 0.882 at T4).

#### Acceptability and usability

To explore participant experience, we administered a self-designed Self-Assessment Scale of Overall Efficacy and Satisfaction (SASE). This exploratory instrument comprised 7 items rated on a 4-point Likert scale (1 = strongly disagree, 4 = strongly agree) to assess general acceptability, plus one open-ended question for qualitative feedback. The SASE was voluntarily available within the intervention platform at any time during the study. It was completed by 44 of the 97 participants in the MVPTC group (response rate: 45.4%). As an unvalidated, study-specific measure, its results are reported for descriptive purposes to complement the primary outcomes. The complete SASE is provided in Supplementary Material S2.

### Statistical analysis

Regarding the differences between groups during the baseline period, independent sample t-tests were used for continuous variables, and Welch’s t-tests were used when variances were uneven. The chi-square test was used for categorical variables, and the Fisher’s exact test was used when the expected frequency was less than 5. All baseline comparisons were descriptive analyses as randomization ensured balance between groups. All analyses followed the intention-to-treat principle, including all randomized participants with baseline data. Linear Mixed Effects Models (LMM) were fitted using the lme4 package in R. The model specification for each outcome was: Y_ij = β₀ + β1*Group_i + Σβ_k*Time_jk + Σβ_m*(Group_i × Time_jm) + u_i + ε_ij.

where Y_ij is the outcome score for participant i at time j, Group_i is a dummy variable (0 = Control, 1 = Intervention), Time_jk are dummy variables representing assessment time points (k = 2, 3, 4, with T1 as reference), u_i is the random intercept for participant i [u_i ~ N(0, σ²_u)], and ε_ij is the residual error [ε_ij ~ N(0, σ²_ε)]. Models were estimated using Restricted Maximum Likelihood (REML). Type III tests with Kenward-Roger degrees of freedom were used for hypothesis testing on fixed effects. Model comparisons using Akaike Information Criterion (AIC) and Bayesian Information Criterion (BIC) indicated that an unstructured covariance matrix provided superior fit compared to compound symmetry for all primary outcomes. A pre-specified hierarchical testing strategy was employed. Depressive symptoms (SDS) and anxiety symptoms (SAS) were designated as co-primary outcomes and tested with a Bonferroni-corrected threshold of *P* < 0.025 (0.05/2). Psychological resilience (CD-RISC) was a pre-specified secondary outcome tested at *P* < 0.05. Sleep quality (ISI), coping style (SCSQ), and perceived social support (PSSS) were analyzed as exploratory outcomes without further correction to preserve statistical power for hypothesis generation; exact P-values and effect sizes are reported for these outcomes. Significant Group * Time interactions were followed by pairwise comparisons at each time point using the emmeans package, with Bonferroni adjustment for multiple comparisons within each outcome. Cohen’s d was calculated for between-group differences at each time point following Feingold’s recommendations for repeated measures designs [60].

To assess the robustness of primary findings, we conducted several sensitivity analyses including complete-case analysis, assessment of clinical significance, evaluation of alternative covariance structures, and outlier analysis using the median absolute deviation method. All analyses were conducted using R (version 4.3.2) and SPSS (version 23.0). All tests were two-sided, with *P* < 0.05 considered statistically significant unless otherwise specified for multiple comparisons.

## Results

### Study flow and participant characteristics

A total of 204 participants with mild to moderate depression or anxiety were enrolled and randomized to either the intervention group (*n* = 97) or the control group (*n* = 107). Baseline characteristics were well-balanced between groups, with no statistically significant differences in any sociodemographic or clinical variables (all *P* > 0.05; Table 1). The overall sample had a mean age of 30.68 years (SD = 7.61), with a predominance of female participants (73.5%). This gender distribution aligns with established epidemiological patterns showing higher prevalence of affective disorders among females (female-to-male ratio typically ranging from 1.5:1 to 2:1) [61]. Participants were highly educated, with 94.1% having college education or above. The majority (94.1%) were either students or employed, indicating an actively engaged population. At enrollment, participants exhibited clinically significant symptoms, with mean scores indicating moderate depression (SDS: 61.65 ± 6.21) and anxiety (SAS: 51.70 ± 7.49). The sample also showed reduced psychological resilience (CD-RISC: 1.99 ± 0.60) and mild insomnia symptoms (ISI: 10.08 ± 5.05). Complete baseline characteristics are presented in Table 1.

**Table 1:** Demographic and clinical variables of participants at baseline

Characteristics	Intervention group (*n* = 97)	Control group (*n* = 107)	*P* value
**Sociodemographic features**			
**Gender**,** n(%)**			0.34
Male	29 (29.90)	25 (23.36)	
Female	68 (70.10)	82 (76.64)	
**Age**,** mean (SD)**	29.75 (7.88)	31.71 (7.20)	0.07
**Marriage**,** n(%)**			0.34
Unmarried	50 (51.55)	66 (61.68)	
Married	43 (44.33)	38 (35.51)	
Divorced/Widowed	4 (4.12)	3 (2.80)	
**Education**,** n(%)**			0.63
Middle school/Below	0 (0.00)	1 (0.93)	
Senior high school	5 (5.15)	6 (5.61)	
College/Above	92 (94.85)	100 (93.46)	
**Employment**, ***n*(%)**			0.45
Student/ Employed	92 (94.85)	100 (93.46)	
Unemployed/ Retired	5 (5.15)	7 (6.54)	
**Clinical characteristics**,** mean (SD)**			
**SDS**^**a**^	62.06 (5.55)	61.29 (6.77)	0.37
**SAS**^**b**^	52.19 (7.66)	51.27 (7.34)	0.38
**CD-RISC**^**c**^	1.96 (0.58)	2.01 (0.61)	0.58
CD-RISC(toughness)	1.89 (0.64)	1.98 (0.58)	0.36
CD-RISC(self-improvement)	2.11 (0.58)	2.10 (0.73)	0.98
CD-RISC(optimism)	2.01 (0.77)	1.77 (0.79)	0.06
**SCSQ**^**d**^	-0.02 (1.25)	0.02 (1.17)	0.81
**ISI**^**e**^	9.89 (4.93)	10.24 (5.18)	0.62
**PSSS**^**f**^	51.39 (14.62)	49.68 (15.22)	0.42
PSSS(family support)	16.99 (6.76)	16.17 (6.93)	0.40
PSSS(friend support)	18.02 (5.56)	16.92 (5.91)	0.18
PSSS(other support)	16.38 (5.68)	16.58 (6.24)	0.81

^a^SDS: The Chinese version of the Self-Rating Depression Scale

^b^SAS: The Chinese version of the Self-Rating Anxiety Scale

^c^CD-RISC: The Connor-Davidson Resilience Scale

^d^SCSQ: Simplified Coping Style Questionnaire including the positive subdimension and the negative subdimension. The SCSQ score was calculated by the Z _positive score_ minus the Z _negative score_

^e^ISI: Insomnia Severity Index

^f^PSSS: Perceived Social Support Scale

### Attrition

**Fig. 1:**
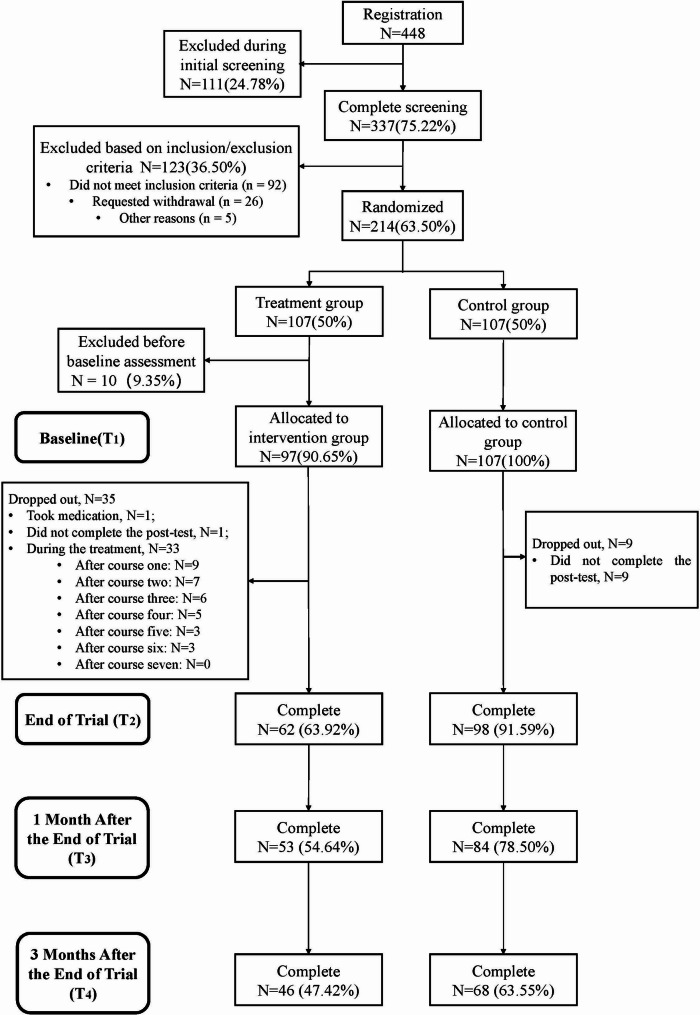
Flow diagram. Note: ITT: Intention-to-Treat analysis included all randomly assigned participants. In the intervention group, 10 participants were excluded prior to commencing the intervention due to failure to complete the baseline assessment on time; they are included in the randomized count but not in the intervention commencement or ITT analysis counts

Participants’ completion and dropout status across assessment time points are presented in Table S1 and Fig. 1. In the intervention group, A total of 62 participants (63.92%) completed the intervention and the post-intervention assessment. In addition, 33 participants dropped out during the treatment (e.g., 9 after the first course, 7 after the second), while 1 participant didn’t complete the post-test, and another one started to take medication during the intervention. By the end of the intervention (T_2_), 62 participants (63.92%) remained. 53 participants (54.64%) remained at one month (T_3_), and 46 (47.42%) remained at three months (T_4_).

In the control group, 9 participants (8.41%) dropped out, all due to not completing the post-test. By the end of the intervention (T_2_), 98 participants (91.59%) remained. At the one-month follow-up, 84 participants (78.50%) remained, and 68 (63.55%) remained at three months post-intervention.

Of the randomized participants, 78.43% (160/204) went on to provide complete data at T2 representing an overall attrition rate of 21.57%. Attrition was not equal between the arms and was greater among the intervention group (36.08% vs. 8.41%; *χ2* = 228.14; *P* < 0.01). However, independent samples *t*-tests and chi-square analyses revealed no significant baseline differences between study completers and dropouts across most sociodemographic and clinical variables(gender, *χ2* = 0.82, *P* = 0.44; education, *χ2* = 1.37, *P* = 0.51; employment, *χ2* = 3.51, *P* = 0.70; marriage, *χ2* = 1.12, *P* = 0.57; SDS, *t*= -0.40, *P* = 0.69; SAS, *t* = -0.96, *P* = 0.34; SCSQ, *t* = -0.22, *P* = 0.83; PSSS, *t* = 0.73, *P* = 0.47; ISI, *t* = 0.31, *P* = 0.71), except on age (dropouts: mean age 28.05 years, SD 4.52; completers: mean age 31.41 years, SD 8.12; t = 2.63; *P* < 0.01) and on the CD-RISC (dropouts: mean score 0.20, SD 0.76; completers: mean score 1.97, SD 0.58; *t* = 16.67; *P* < 0.01). It suggests that younger participants and those with poorer psychological resilience are more likely to drop out.

### Use and acceptability

Table 2 summarizes video engagement data for the subset of participants (*n* = 34) for whose verifiable viewing logs were available due to intermittent instability of the digital platform. Viewing duration was recorded by the platform in whole minutes, with values rounded to the nearest integer. Across all eight micro-video courses, the total viewing time had a mean of 48.3 min (*SD* = 19.2) and a median of 42.5 min (interquartile range = 33.2–57.5), which was longer the total video duration of 34.1 min. Individual total viewing times ranged from 22 to 92 min. The distribution of total viewing time was right-skewed, with several high-end outliers (e.g., values above 80 min). No statistically significant Pearson correlations were observed between overall viewing duration and symptom changes (SDS, *r* = -0.09, *P* = 0.67; SAS, *r* = 0.16, *P* = 0.43; CD, *r* = 0.32, *P* = 0.15; ISI, *r* = 0.07, *P* = 0.72).

**Table 2:** Video engagement data for each micro-video course (Mean ± SD)

	Duration of micro-video (min)	Average viewing time (min)	Median (min)
Course one	3.47	4.44 ± 2.60	3
Course two	3.48	4.50 ± 3.10	3
Course three	4.70	6.65 ± 4.24	4.5
Course four	4.93	7.12 ± 4.30	5
Course five	4.10	5.38 ± 2.85	5
Course six	4.07	5.38 ± 3.42	4
Course seven	4.52	7.91 ± 6.20	5
Course eight	4.83	6.88 ± 5.15	5
Total	34.10	48.26 ± 19.16	42.5

**Fig. 2:**
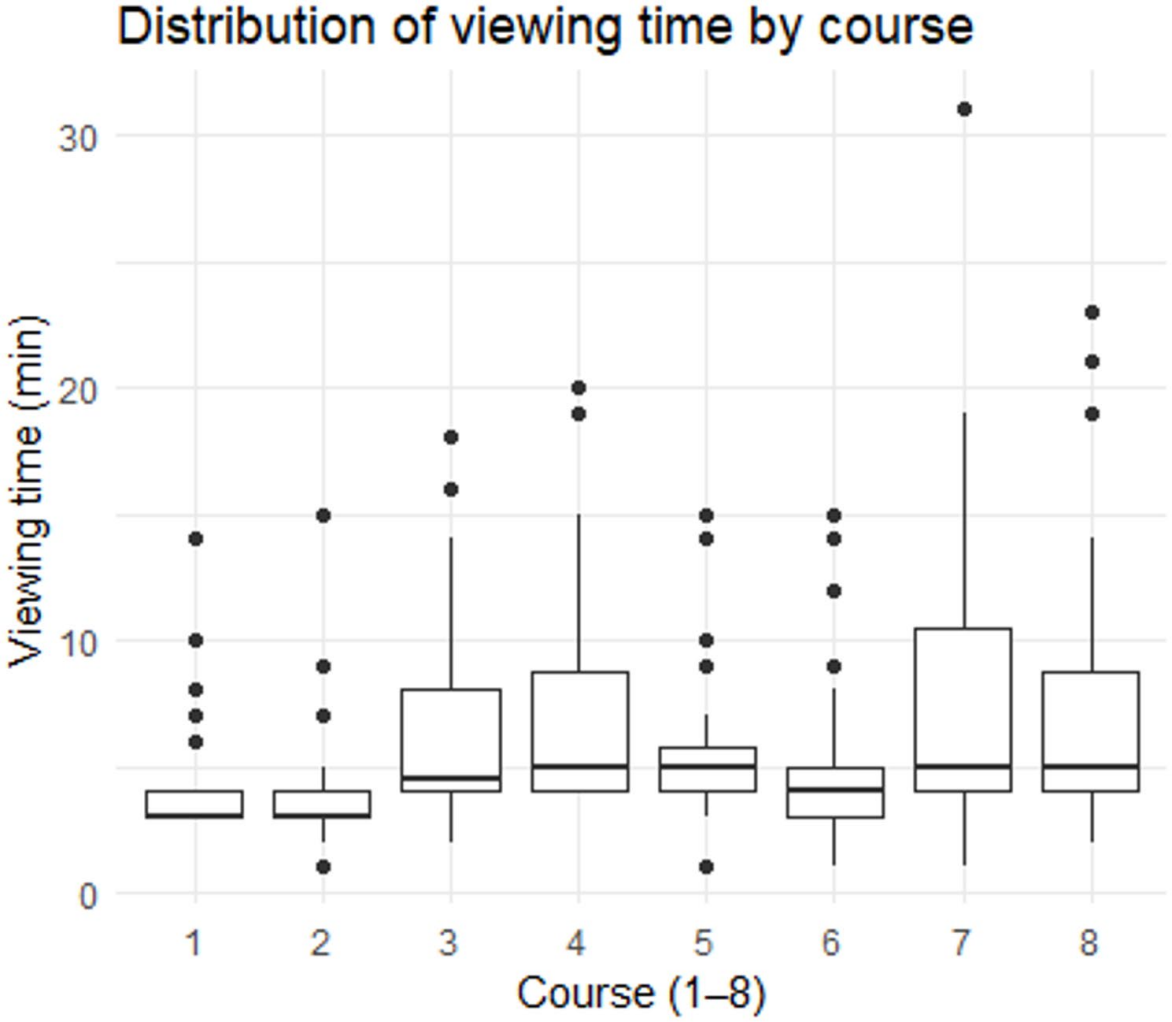
Boxplots of the distribution of viewing time for each course

Table 2 summarizes video engagement data for each course. For individual courses, median viewing times were close to or exceeded the video duration. Notably, variability in viewing time differed across courses, with Course seven showing the highest standard deviation (6.20 min) and the presence of multiple extreme outliers in its boxplot (Fig. 2), indicating heterogeneous engagement patterns for this specific content.

Figure 2 illustrates the distribution of viewing time for each course using boxplots. Median viewing times were close to or slightly longer than the original video duration across courses. The upper whiskers and outliers indicated a right-skewed distribution for most courses, with several notable outliers suggesting instances of either very prolonged engagement or potential passive playback.

**Fig. 3:**
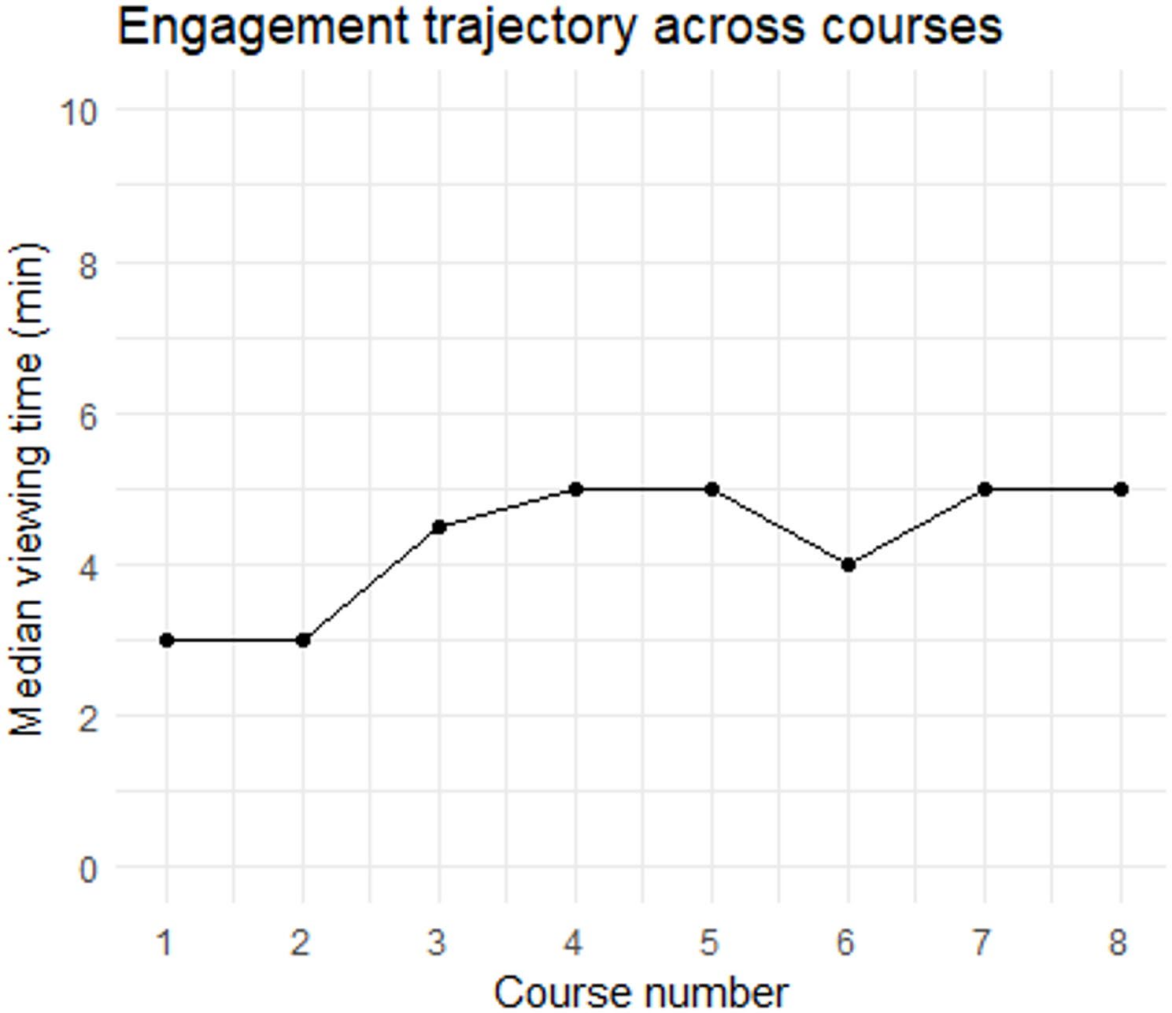
Engagement trajectory across the eight courses

Figure 3 presents the engagement trajectory across the eight courses based on median viewing time. Median engagement remained relatively stable across courses.

Overall, participants rated the intervention as acceptable (mean score = 3.06, SD = 0.41, on a 0–4 scale, *n* = 44). Those in the MVPTC group reported high satisfaction across several specific domains: simplicity and intelligibility of the applet (mean = 3.57, SD = 0.50), acceptance of the micro-video format (mean = 3.36, SD = 0.61), relevance of homework to daily life (mean = 3.36, SD = 0.69), comprehensibility of the content (mean = 3.25, SD = 0.69), and perceived helpfulness (mean = 3.20, SD = 0.63). However, when compared to face-to-face psychotherapy or more interactive treatments, participants still expressed a preference for face-to-face delivery (mean = 1.89, SD = 0.81, reverse-scored) and for skills being taught in person (mean = 2.82, SD = 0.76, reverse-scored).

### Model specification and sensitivity analyses

#### Model fit and covariance structure selection

For all primary outcomes (depression [SDS], anxiety [SAS], and resilience [CD-RISC]), model fit was assessed using the Akaike Information Criterion (AIC) and Bayesian Information Criterion (BIC). The unstructured covariance structure provided a better fit than the compound symmetry structure (SDS: AIC 4183.18 vs. 4185.18; SAS: 4029.31 vs. 4031.31; CD-RISC: 773.20 vs. 775.20). Therefore, the unstructured covariance model was used for all final linear mixed model analyses (see Supplementary Table S2 for detailed fit statistics).

#### Sensitivity analyses

Several sensitivity analyses confirmed the robustness of the primary findings (Supplementary Table S3):

Complete-case analysis: Results based on participants with complete data at all four time points (*n* = 104, 51.0% of the sample) were consistent with the intention-to-treat analysis.

Clinical significance: At the 3-month follow-up (T4), the intervention group exhibited higher remission rates than the control group (depression: 67.4% vs. 50.0%; anxiety: 76.1% vs. 58.8%).

Covariance structure comparison: Analyses conducted using the compound symmetry covariance structure yielded substantively identical results to the primary analyses.

Outlier analysis: The proportion of outliers across all variables at each time point was below 2%, indicating satisfactory data quality and stability of the estimates.

## Treatment outcomes

### Outcome measures

**Fig. 4:**
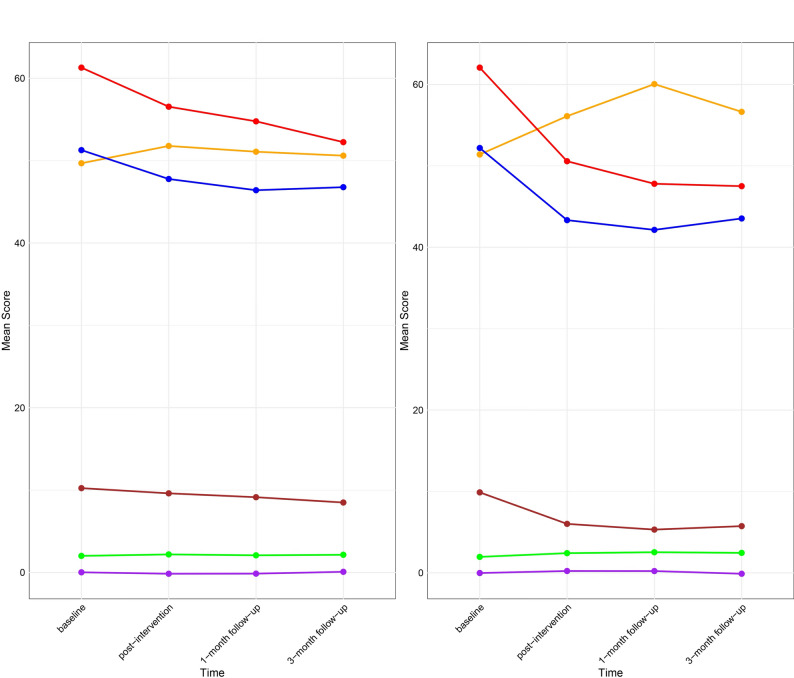
Statistical analyses for outcomes at different time points. (**a**) Control group (**b**) Intervention group

Table 3; Fig. 4 shows the means and standard deviations of various psychological measures across four time points: baseline (T_1_), post-intervention (T_2_), one-month follow-up (T_3_), and three-month follow-up (T_4_). The figure legend presents a simplified key for interpreting the variables depicted in the graph. Each variable is assigned a unique color for clear differentiation: CD (green), PSSS (orange), SCSQ (purple), ISI (red), SAS (blue), and SDS (red). To facilitate direct comparison between groups, Fig. 4 presents the trajectories of the three primary outcomes separately: depressive symptoms (SDS, panel a), anxiety symptoms (SAS, panel b), and psychological resilience (CD-RISC, panel c). Each panel displays the estimated marginal means (with standard error bars) for both the intervention and waitlist control groups across the four assessment time points. As shown, the intervention group demonstrated steeper declines in SDS and SAS scores and greater increases in CD-RISC scores compared to the control group (Supplementary Figure S1).

**Table 3:** Statistical analyses for outcomes at different timepoints

	Timepoints			
	T_1_(*n* = 204), mean(SD)	T_2_(*n* = 160), mean(SD)	T_3_(*n* = 137), mean(SD)	T_4_(*n* = 104), mean(SD)
**Control group**,** mean (SD)**	(*n* = 107)	(*n* = 98)	(*n* = 84)	(*n* = 68)
SDS	61.29(6.77)	56.54(9.05)	54.77(9.94)	52.24(12.52)
SAS	51.27(7.34)	47.77(8.27)	46.41(9.27)	46.77(10.43)
CD-RISC	2.01(0.61)	2.19(0.71)	2.08(0.74)	2.14(0.72)
CD-RISC(toughness)	1.98(0.58)	2.11(0.73)	2.09(0.73)	2.23(0.78)
CD-RISC(self-improvement)	2.10(0.73)	2.32(0.78)	2.15(0.83)	2.18(0.79)
CD-RISC(optimism)	1.77(0.79)	2.18(0.82)	1.91(0.81)	1.80(0.83)
SCSQ	0.02(1.17)	-0.15(1.08)	-0.14(1.08)	0.08(0.90)
PSSS	49.68(15.22)	51.78(16.30)	51.08(16.86)	50.60(17.88)
PSSS(family support)	16.17(6.93)	17.01(7.11)	16.28(7.14)	15.51(8.53)
PSSS(friend support)	16.92(5.91)	17.34(6.03)	17.34(6.12)	18.00(6.15)
PSSS(other support)	16.58(6.24)	17.43(6.41)	17.46(6.16)	17.09(6.66)
ISI	10.24(5.18)	9.61(5.75)	9.14(5.34)	8.49(4.96)
**Intervention group**,** mean (SD)**	(*n* = 97)	(*n* = 62)	(*n* = 53)	(*n* = 46)
SDS	62.06(5.55)	50.56(9.43)	47.79(11.17)	47.50(10.71)
SAS	52.19(7.66)	43.33(8.26)	42.13(8.15)	43.53(10.43)
CD-RISC	1.96(0.58)	2.41(0.68)	2.53(0.75)	2.45(0.68)
CD-RISC(toughness)	1.89(0.64)	2.36(0.72)	2.48(0.77)	2.49(0.70)
CD-RISC(self-improvement)	2.11(0.58)	2.51(0.74)	2.59(0.81)	2.43(0.78)
CD-RISC(optimism)	2.01(0.77)	2.27(0.63)	2.58(0.77)	2.35(0.90)
SCSQ	-0.02(1.25)	0.23(1.07)	0.22(1.22)	-0.11(1.15)
PSSS	51.39(14.62)	56.10(16.41)	60.04(14.16)	56.63(14.90)
PSSS(family support)	16.99(6.76)	19.10(6.93)	19.88(6.19)	17.63(9.75)
PSSS(friend support)	18.02(5.55)	19.45(6.05)	20.57(5.22)	19.61(5.45)
PSSS(other support)	16.38(5.68)	17.55(6.40)	19.59(5.71)	19.39(5.35)
ISI	9.89(4.93)	6.02(4.35)	5.31(3.98)	5.74(4.96)

### Intention-to-treat analysis

Table 4 presents the descriptive statistics (means and standard deviations) for all outcome measures across the four assessment time points (T1-T4) for both the intervention and control groups.

**Table 4:** Linear mixed model results: estimated means, standard errors, and between-group differences at each assessment time point

Outcome Measure	Time	Intervention Group Mean (SE)	Control Group Mean (SE)	Between-Group Difference (95% CI)	*P* value	Cohen’s d
SDS	T1	62.06 (0.56)	61.29 (0.65)	0.78 (-1.74, 3.30)	0.54	-0.11
	T2	50.56 (1.20)	56.54 (0.91)	5.96 (3.12, 8.80)	< 0.01	0.87
	T3	47.79 (1.53)	54.77 (1.08)	6.82 (3.74, 9.90)	< 0.01	0.99
	T4	47.50 (1.58)	52.24 (1.52)	5.49 (1.88, 9.10)	< 0.01	0.80
SAS	T1	52.19 (0.78)	51.27 (0.71)	-0.92 (-3.26, 1.42)	0.44	-0.15
	T2	43.33 (1.05)	47.77 (0.84)	4.36 (1.89, 6.83)	< 0.01	0.71
	T3	42.13 (1.12)	46.41 (1.01)	4.64 (1.85, 7.43)	< 0.01	0.75
	T4	43.53 (1.54)	46.77 (1.26)	3.88 (0.90, 6.86)	0.01	0.63
CD-RISC	T1	1.96 (0.06)	2.01 (0.06)	0.05 (-0.16, 0.26)	0.64	0.13
	T2	2.41 (0.09)	2.19 (0.07)	-0.20 (-0.42, 0.02)	0.08	-0.48
	T3	2.53 (0.10)	2.08 (0.08)	-0.40 (-0.64, -0.16)	< 0.01	-0.97
	T4	2.45 (0.10)	2.14 (0.09)	-0.35 (-0.59, -0.11)	< 0.01	-0.84
CD-RISC (toughness)	T1	1.89 (0.07)	1.98 (0.06)	0.09 (-0.13, 0.31)	0.43	0.21
	T2	2.36 (0.09)	2.11 (0.07)	-0.22 (-0.44, 0.00)	0.05	-0.52
	T3	2.48 (0.11)	2.09 (0.08)	-0.36 (-0.61, -0.11)	< 0.01	-0.83
	T4	2.49 (0.10)	2.23 (0.09)	-0.31 (-0.56, -0.06)	0.01	-0.70
CD-RISC (self-improvement)	T1	2.11 (0.06)	2.10 (0.07)	0.00 (-0.20, 0.20)	0.98	-0.01
	T2	2.51 (0.09)	2.32 (0.08)	-0.18 (-0.42, 0.06)	0.14	-0.38
	T3	2.59 (0.11)	2.15 (0.09)	-0.41 (-0.68, -0.14)	< 0.01	-0.87
	T4	2.43 (0.11)	2.18 (0.10)	-0.34 (-0.62, -0.06)	0.01	-0.72
CD-RISC (optimism)	T1	2.01 (0.08)	1.77 (0.08)	-0.24 (-0.50, 0.02)	0.07	-0.39
	T2	2.27 (0.08)	2.18 (0.08)	-0.09 (-0.34, 0.16)	0.48	-0.15
	T3	2.58 (0.11)	1.91 (0.09)	-0.60 (-0.88, -0.32)	< 0.01	-1.00
	T4	2.35 (0.13)	1.80 (0.10)	-0.56 (-0.88, -0.24)	< 0.01	-0.95
SCSQ	T1	-0.02 (0.13)	0.02 (0.11)	0.04 (-0.28, 0.36)	0.80	0.05
	T2	0.23 (0.14)	-0.15 (0.11)	-0.42 (-0.78, -0.06)	0.02	-0.58
	T3	0.22 (0.17)	-0.14 (0.12)	-0.38 (-0.76, 0.00)	0.04	-0.53
	T4	-0.11 (0.17)	0.08 (0.11)	0.09 (-0.31, 0.49)	0.63	0.13
ISI	T1	9.89 (0.50)	10.24 (0.50)	0.35 (-1.08, 1.78)	0.62	0.10
	T2	6.02 (0.55)	9.61 (0.58)	3.39 (1.66, 5.12)	< 0.01	0.97
	T3	5.31 (0.55)	9.14 (0.58)	3.76 (1.99, 5.53)	< 0.01	1.08
	T4	5.74 (0.73)	8.49 (0.60)	2.44 (0.57, 4.31)	0.01	0.70
PSSS	T1	51.39 (1.48)	49.68 (1.47)	-1.72 (-6.12, 2.68)	0.44	-0.17
	T2	56.10 (2.08)	51.78 (1.65)	-3.81 (-8.65, 1.03)	0.12	-0.38
	T3	60.04 (1.95)	51.08 (1.84)	-6.46 (-11.48, -1.44)	0.01	-0.64
	T4	56.63 (2.20)	50.60 (2.17)	-4.99 (-10.41, 0.43)	0.07	-0.50
PSSS (other)	T1	16.38 (0.58)	16.58 (0.60)	0.20 (-1.58, 1.98)	0.82	0.05
	T2	17.55 (0.82)	17.43 (0.65)	0.03 (-1.98, 2.04)	0.97	0.01
	T3	19.59 (0.78)	17.46 (0.67)	-1.31 (-3.28, 0.66)	0.19	-0.34
	T4	19.39 (0.79)	17.09 (0.81)	-2.06 (-4.14, 0.02)	0.05	-0.53
PSSS (family)	T1	16.99 (0.69)	16.17 (0.67)	-0.82 (-2.89, 1.25)	0.43	-0.18
	T2	19.10 (0.88)	17.01 (0.72)	-1.71 (-3.93, 0.51)	0.13	-0.37
	T3	19.88 (0.85)	16.28 (0.78)	-2.55 (-4.84, -0.26)	0.03	-0.55
	T4	17.63 (1.44)	15.51 (1.03)	-1.63 (-4.08, 0.82)	0.19	-0.35
PSSS (friend)	T1	18.02 (0.56)	16.92 (0.57)	-1.10 (-2.76, 0.56)	0.19	-0.28
	T2	19.45 (0.77)	17.34 (0.61)	-2.13 (-4.00, -0.26)	0.02	-0.55
	T3	20.57 (0.72)	17.34 (0.67)	-2.62 (-4.50, -0.74)	0.01	-0.68
	T4	19.61 (0.80)	18.00 (0.75)	-1.31 (-3.32, 0.70)	0.20	-0.34

Note Values are estimated means (standard errors) based on linear mixed models. Between-group differences represent control group minus intervention group. Positive differences for SDS and SAS indicate greater symptom reduction in the intervention group; negative differences for CD-RISC and its subscales indicate greater resilience improvement in the intervention group. For SCSQ, negative differences indicate more positive coping strategies in the intervention group. For ISI, positive differences indicate greater sleep improvement in the intervention group. For PSSS and its subscales, negative differences indicate greater perceived social support in the intervention group. CI = confidence interval; SE = standard error. T1 = baseline, T2 = post-intervention, T3 = 1-month follow-up, T4 = 3-month follow-up

#### Primary outcomes

For SDS, the results of linear mixed model showed that the group × time effect was significant, *F*_3, 1218_ = 10.18, *P* < 0.01, *η*^*2*^ = 0.10. The linear mixed model revealed SDS scale scores were compared between intervention group and control group at four time points. Significant improvements were observed in the intervention group compared to the control group at T_2_ (*E* = 5.96, *P* < 0.01, Cohen’s *d* = 0.87), T3 (*E* = 6.82, *P* < 0.01, Cohen’s *d* = 0.99), and T_4_ (*E* = 5.49, *P* < 0.01, Cohen’s *d* = 0.80).

For SAS, the results of linear mixed model showed that the group × time effect was significant, *F*_3, 1218_ = 7.20, *P* < 0.01, *η*^*2*^ = 0.13. The intervention group showed significant reductions at T_2_ (*E* = 4.36, *P* < 0.01, Cohen’s *d* = 0.71), T_3_ (*E* = 4.64, *P* < 0.01, Cohen’s *d* = 0.75), and T_4_ (*E* = 3.88, *P* = 0.01, Cohen’s *d* = 0.63). Those results indicated severer depression and anxiety symptoms in control group than intervention group, which as well showed that the treatment works at post-intervention and follow-ups. All between-group differences for the primary outcomes (SDS and SAS) remained statistically significant after applying the Bonferroni-corrected threshold of *P* < 0.025 (Table 4).

Beyond group-mean comparisons, we evaluated the clinical significance of intervention effects using three established metrics (Table 5). For anxiety symptoms, a significantly higher proportion of intervention participants achieved reliable improvement (63.0% vs. 34.3% in controls, *χ²* = 7.93, *P* = 0.005) and shifted from mild-to-moderate severity at baseline to the normal range at follow-up (41.3% vs. 19.4%, *χ²* = 5.41, *P* = 0.020). For depression, similar trends were observed, with 69.6% of intervention participants showing reliable improvement versus 52.2% in controls (*P* = 0.100), and 60.9% moving to the normal severity range compared to 40.3% (*P* = 0.050). Clinical remission rates (scores below clinical cut-offs) were consistently higher in the intervention group for both depression (67.4% vs. 50.7%) and anxiety (76.1% vs. 59.7%), though these differences did not reach statistical significance (both *P* > 0.10).

**Table 5:** Clinical significance outcomes at 3-month follow-up (T4)

Outcome measure	Control group (*n* = 68)	Intervention group (*n* = 46)	χ²	*P* value
SDS				
Reliable improvement	35 (52.2%)	32 (69.6%)	2.71	0.100
Severity shift to normal*	24 (40.3%)	28 (60.9%)	3.83	0.050
Clinical remission (SDS < 53)	34 (50.7%)	31 (67.4%)	2.45	0.118
SAS				
Reliable improvement	23 (34.3%)	29 (63.0%)	7.93	0.005
Severity shift to normal†	15 (19.4%)	19 (41.3%)	5.41	0.020
Clinical remission (SAS < 50)	40 (59.7%)	35 (76.1%)	2.59	0.108

Note. Reliable improvement = Reliable Change Index > 1.96. Severity shift to normal = movement from mild or moderate severity at baseline to the normal range at T4

*SDS severity categories: Normal (≤ 53), Mild (53–62), Moderate (63–72)

†SAS severity categories: Normal (≤ 50), Mild (50–59), Moderate (60–69)

#### Secondary outcome

For CD-RISC, the group × time effect was significant, *F*_3, 1218_ = 8.01, *P* < 0.01, *η*^*2*^ = 0.27. Improvements were significant for overall resilience at T_3_ (*E* = -0.40, *P* < 0.01, Cohen’s *d* = -0.97) and T_4_ (*E* = -0.35, *P* < 0.01, Cohen’s *d* = -0.84). Subdomains (i.e., toughness, self-improvement, and optimism) showed significant improvements in the intervention group vs. the control group from T_3_ to T_4_ (*P* ranging from < 0.01 to 0.01, | Cohen’s *d |* ranging from 0.01 to 1.00). Those results showed that resilience in the intervention group was higher than those in the control group at follow-ups, indicating benefits on resilience of the treatment.

#### Exploratory outcomes

For ISI, the interaction effect was significant, *F*_3, 1218_ = 7.40, *P* < 0.01, *η*^*2*^ = 0.25. Significant improvements were observed in the intervention group compared to the control group at T_2_ (*E* = 3.39, *P* < 0.01, Cohen’s *d* = 0.97), T_3_ (*E* = 3.76, *P* < 0.01, Cohen’s *d* = 1.08), and T_4_ (*E* = 2.44, *P* = 0.01, Cohen’s *d* = 0.70), which showed that the treatment was effective on sleeping problems.

For SCSQ, the interaction effect was significant, *F*_3, 1218_ = 4.69, *P* < 0.01, *η*^*2*^ = 0.91. Significant differences occurred at T_2_ (*E* = -0.42, *P* = 0.02, Cohen’s *d* = -0.58) and T_3_ (*E* = -0.38, *P* = 0.04, Cohen’s *d* = − 0.53), reflecting better coping strategies in the intervention group. However, this difference disappeared at T4, indicating a short influence on coping style.

For PSSS, there was no interaction effect, *F*_3, 1218_ = 1.37, *P* = 0.25, *η*^*2*^ = 0.22. Significance was only observed at T_3_ (*E* = -6.46, *P* = 0.01, Cohen’s *d* = -0.64), showing that more supports were perceived by the intervention group at a short time after the treatment, especially family support (*E* = -2.55, *P* = 0.03, Cohen’s *d* = -0.55) and friend support (*E* = -2.62, *P* = 0.01, Cohen’s *d* = -0.68).

## Discussion

### Principal results

The Micro-Video Psychological Training Camp (MVPTC), a fully automated, integrative digital intervention hosted on WeChat, demonstrated significant efficacy in reducing symptoms of depression and anxiety while enhancing psychological resilience in a Chinese sample with mild-to-moderate symptoms. For primary outcomes, the intervention group showed sustained reductions in depressive symptoms (SDS: Cohen’s *d* = 0.80–0.99, *P* < 0.01) across post-intervention (T2), 1-month (T3), and 3-month follow-ups (T4), with stable large effect sizes. Anxiety symptoms (SAS) also decreased significantly (Cohen’s *d* = 0.63–0.75, *P* ≤ 0.01), and moderate-to-large effects for anxiety symptoms. These effect sizes are comparable to those reported in meta-analyses of internet-based Cognitive Behavioral Therapy (iCBT) for depression and anxiety (e.g., *d* ≈ 0.7-1.0) [27, 62], yet they are understandably somewhat smaller than the effects typically observed in face-to-face CBT or guided digital interventions [63]. This difference likely reflects the fully self-guided, low-intensity nature of the MVPTC, which trades some efficacy for greater scalability and accessibility—a rational trade-off in a context of severe resource constraints. The effect sizes for anxiety were moderately smaller than for depression, suggesting content could be further optimized. Future versions should integrate more targeted exposure exercises and cognitive techniques addressing core anxiety mechanisms (e.g., intolerance of uncertainty) [64, 65].

The most robust secondary outcome was the improvement in psychological resilience (CD-RISC), with large effect sizes (CD-RISC: Cohen’s *d* = -0.84 to -0.97, *η²* = 0.27, *P* < 0.01) emerging at follow-ups. Resilience—the capacity to adapt to stress—is a positive resource that may protect against future relapse [49, 66]. The substantial improvement in resilience suggests that the MVPTC’s integrative framework successfully fostered a broader skill set (emotional regulation, cognitive flexibility, perceived support) beyond symptom reduction, aligning with a preventive and mental health promotion paradigm.

### Mechanistic insights and exploratory outcomes

The exploratory outcomes offer preliminary insights into the intervention’s mechanisms and scope. The strong, durable effect on insomnia severity (ISI) is consistent with the transdiagnostic model, as sleep disturbances are tightly linked to both depressive rumination and anxious arousal, forming a well-established bidirectional maintenance cycle [67]. The intervention likely disrupted this cycle through techniques promoting cognitive de-arousal (mindfulness) and behavioral regulation.

In contrast, improvements in coping styles (SCSQ) and perceived social support (PSSS) were transient. Transient effects were observed for coping strategies (SCSQ: T2-T3 Cohen’s *d*= -0.53 to -0.58, *P* < 0.05), with no significant differences at T4, and PSSS showed only temporary increases in family/friend support at T3 (Cohen’s *d* = -0.55 to -0.68, *P* < 0.05) without overall group interaction (*F* = 1.37, *P* = 0.25). This pattern suggests that while participants initially learned and applied new coping strategies and felt a short-term boost in support, maintaining these changes in the long term without ongoing structure or reinforcement proved challenging. This underscores a limitation of the current brief, self-guided format for effecting durable change in complex behavioral and interpersonal domains. It highlights the potential need for periodic booster sessions, integration of peer support elements, or more intensive skills training modules to solidify these gains.

### The integrative approach: synergy and challenge

This study adopted an integrative framework combining CBT, DBT/mindfulness, and IPT principles, predicated on the theoretical synergy of concurrently targeting the core, interacting maintenance mechanisms of comorbid depression and anxiety [68]. The positive outcomes across multiple domains provide preliminary support for this synergistic model, consistent with meta-analytic evidence for transdiagnostic approaches [15]. However, an integrative approach also presents challenges. Presenting multiple therapeutic frameworks within a brief intervention risks creating cognitive load, potentially hindering deep skill mastery. Furthermore, inherent tensions exist between modalities—for instance, between CBT’s emphasis on changing thoughts and mindfulness’s emphasis on accepting them. In the MVPTC, we aimed to frame these as complementary (e.g., “acceptance as a foundation for wise change”), but future research should examine how users actually perceive and integrate these skill sets. Optimizing the sequencing, pacing, and psychoeducation of these components is a crucial avenue for development.

### Adherence and dropout

Dropout remains a significant challeng*e* in digital mental health interventions. Melville et al. reported that internet-based psychological treatment programs have a wide range of dropout rates, from 2 to 83%, with a weighted average of 31% [69]. In this study, over 60% of participants completed all eight sessions, a rate comparable to prior automated trials (62.2% retention). Engagement metrics further showed that participants’ median viewing time per video often equaled or exceeded its nominal duration. While this could indicate repeated viewing or prolonged engagement with the content, technical limitations noted below preclude distinguishing this from passive playback (e.g., videos left running). However, attrition followed a distinct pattern: 66.7% of dropouts occurred after the first three sessions, aligning with the common “early disengagement window” in self-guided interventions [70]. This early attrition may be linked to the content of these initial modules, which focused on psychoeducation, emotional identification, regulation, and resilience-building. While essential for establishing a foundation, these abstract skills may not provide the immediate symptom relief some users expect when assessing the intervention’s personal relevance, potentially leading to disengagement. Participants who continued beyond this early window demonstrated stable adherence, underscoring that persisting through the foundational phase is key to program completion.

Dropout patterns likely reflect participant- and intervention-related factors. Younger age and lower psychological resilience predicted attrition, consistent with meta-analytic evidence linking higher dropout rates to youth and emotional vulnerability [71]. Younger participants may struggle with sustained engagement in self-guided formats, while those with limited resilience may lack coping strategies to persist. Treatment-related contributors include mismatched expectations (e.g., preference for face-to-face modalities) and intervention complexity [69].

### Strengths and limitations

This study has several strengths. This study has several strengths, including its randomized controlled design, use of an intent-to-treat analytic approach, long-term follow-up, the development of a novel, culturally-situated intervention for a high-need population, its integrative psychotherapeutic framework targeting core transdiagnostic mechanisms, and the inclusion of psychological resilience as a key outcome assessing long-term protective factors.

The limitations must be thoughtfully considered. First and most critically, the use of a wait-list control group, while common, means that non-specific factors—such as the attention received from research participation, assessment reactivity, participant expectations, and natural fluctuation—cannot be disentangled from the specific effects of the integrative psychotherapy content. This substantially limits causal inference regarding the active ingredients of the MVPTC. Future trials require an attention-matched active control condition (e.g., psychoeducational videos) to isolate the specific efficacy of the therapeutic skills taught. Second, generalizability is constrained by the sample, which was predominantly young, female, highly educated, and smartphone-literate. Additionally, the relatively high dropout rate and underrepresentation of older adults and individuals with more severe symptoms further limit the applicability of the findings. While this reflects the early-adopter demographic for digital health in China and was shaped by pragmatic recruitment constraints, it necessitates caution in extending findings to older, less educated, or more severe populations. Moreover, the requirement for participants to be free of regular treatment for six months prior to enrollment means our results primarily apply to individuals with mild-to-moderate symptoms who are not in active care. Third, reliance on self-report measures introduces the potential for response bias. Furthermore, as is common in behavioral intervention trials, blinding of participants was not feasible, raising the possibility that self-reported improvements were influenced by expectancy effects. Although clinically validated scales were used, the incorporation of blinded clinician-rated interviews or behavioral measures in future research would strengthen the findings. Fourth, the acceptability survey, while informative, was a self-designed measure. Future studies should employ standardized measures of user experience and therapeutic alliance (adapted for digital contexts) for more robust benchmarking. Fifth, the upper age limit was pragmatically extended from 55 to 70 years during recruitment to include one eligible, motivated older participant, constituting a minor protocol deviation. Finally, engagement data were available for only a subset of participants due to intermittent instability of the digital platform, and viewing duration was recorded in rounded whole minutes and does not allow differentiation between repeated viewing, passive exposure (e.g., videos left running), or active attention, which may limit the generalizability of the engagement findings.

## Conclusion and future directions

The Micro-Video Psychological Training Camp (MVPTC) is a promising, evidence-based digital intervention that sustainably alleviates core symptoms while building resilience and improving sleep in adults with mild-to-moderate depression and anxiety in China.

To translate this efficacy into public health impact, a feasible implementation pathway is essential. Key steps include: hosting MVPTC on ubiquitous platforms (e.g., hospital WeChat accounts) to minimize infrastructure costs; adopting a stepped-care oversight model where trained non-specialists manage delivery, backed by mental health professionals for complex cases; embedding the program as a first-line tool in primary care and other community settings with built-in triage protocols; and ensuring ethical implementation through clear severity thresholds and seamless referral pathways to prevent delays in accessing higher-intensity care. By addressing these practical priorities, MVPTC can evolve from a research prototype into a scalable, cost-effective component of an integrated mental healthcare system.

## Supplementary Information

Below is the link to the electronic supplementary material.

Supplementary Material 1

Supplementary Material 2

Supplementary Material 3

Supplementary Material 4

## Data Availability

All relevant data files are available from the corresponding author on reasonable request.

## Abbreviations

RCT: Randomized controlled trial
MVPTC: Micro-Video Psychological Training Camp
CBT: Cognitive Behavioral Therapy
DBT: Dialectical Behavior Therapy
IPT: Interpersonal Psychotherapy
SMHC: Shanghai Mental Health Center
CONSORT: Consolidated Standards Of Reporting Trials
SDS: The Chinese version of the Self-Rating Depression Scale
SAS: The Chinese version of the Self-Rating Anxiety Scale
MINI: Mini-International Neuropsychiatric Interview
CD-RISC: The Connor-Davidson Resilience Scale
PSSS: Perceived Social Support Scale
ISI: Insomnia Severity Index
SCSQ: Simplified Coping Style Questionnaire
SASE: Self-Assessment Scale of the Overall Efficacy and Satisfaction of Participants
LMM: Linear Mixed Effects Models
REML: Restricted Maximum Likelihood
AIC: Akaike Information Criterion
BIC: Bayesian Information Criterion
ITT: Intention-to-Treat principle
SD: Standard deviation
SE: Standard errors
CI: Confidence intervals
