# Supplementary material for: Efficacy of micro-video psychological training camp for reducing depression and anxiety and enhancing resilience: a randomized controlled trial
Source: BMC Psychiatry. 2026 Jan 23;26:173. doi: 10.1186/s12888-026-07807-6 (PMC12910843; doi:10.1186/s12888-026-07807-6)
Supplement: Supplementary file 1 — Supplementary Material 1 [file 12888_2026_7807_MOESM1_ESM.docx]

### ****Supplementary Material S1:****

### ****Complete Intervention Protocol for the Micro-Video Psychological Training Camp (MVPTC)****

**Overview**

The MVPTC is a fully automated, self-guided digital intervention comprising eight sequential modules delivered via a WeChat Mini Program. Each module consists of an animated psychoeducational micro-video (3-5 minutes) narrated by a virtual therapist, followed by a structured self-practice assignment. The intervention is grounded in an integrative psychotherapy framework. Below is the complete, module-by-module outline detailing session objectives, video content, and homework tasks to enable replication.

### ****Module 1: Psychological Stress and Our Reactions****

- **Objective**: To psychoeducate participants on the nature of psychological stress as a common trigger for depressive and anxious symptoms, to build awareness of the interconnected bodily, emotional, cognitive, and behavioral reactions to stress, and to establish the rationale for learning subsequent emotion regulation skills.
- **Video Content Synopsis:**

1. Introduction to the series, stating its goal to help manage stress-related **depressive and anxious feelings** and build resilience.
2. Definition of psychological stress as significant life events (e.g., conflicts, loss, trauma) that disrupt well-being.
3. Description of the body's immediate **bodily sensations and reactions** (e.g., muscle tension, rapid heartbeat, stomach discomfort, feeling "on edge") associated with the "fight-or-flight" or "freeze" response.
4. Focus on subsequent emotional and cognitive reactions most relevant to **depression and anxiety**:

- **Emotional**: Persistent sadness, loss of interest (depressive core), excessive worry, nervousness, irritability (anxious core).
- **Cognitive/Mental**: Negative thinking patterns, self-criticism, difficulty concentrating, catastrophic thoughts.
- **Behavioral**: Social withdrawal, avoidance of activities, agitation, or restlessness.
- **Bodily Sensations:**Fatigue, heaviness in limbs, restlessness, sleep/appetite changes.

1. Explanation of how unmanaged stress can escalate into more persistent **depressive symptoms** (e.g., low mood, fatigue) and **anxiety symptoms** (e.g., chronic tension, sleep problems), affecting daily function.

- **Homework Task:**

**Task 1.1: Psychological Stress and Our Reactions**

- Participants are asked to reflect on a recent stressful event and explicitly identify connections to their mood by recording both the event and their **emotions(e.g., sad, worried), thoughts (e.g., “I can’t cope”)**, behaviors and bodily sensations.

| **Stressful Event** | **Your Reactions (Link to Mood)** |
| --- | --- |
| e.g., An important work deadline | **Emotional**: Anxious, overwhelmed.  **Cognitive**: “I’m going to fail.”  **Behavioral:** Avoided starting, procrastinated by browsing the internet.  **Bodily Sensations：**Tense shoulders, headache |

### ****Module 2: Identifying and Managing Our Emotions****

- **Objective:** Skills adapted from Dialectical Behavior Therapy (DBT) for emotion dysregulation .To teach participants to identify common negative emotions, rate their intensity, understand the sources and functions of emotions, and apply structured strategies for healthy emotional expression and regulation.
- **Video Content Synopsis:**

1. **Review & Introduction:** Recap of the mind-body connection from Module 1, introduction to the skills of this module.
2. **Emotion Identification:**

- Review of common negative emotions: Anxiety, Fear, Anger, Sadness/Depression.
- **Strategy 1: Expand Vocabulary:** Introduce an **Emotion Reference List** (see below) to help name feelings more precisely.
- **Strategy 2: Self-Observation:** Practice asking “What am I feeling right now?” without judgment.

1. **Emotion Assessment:** Teach the skill of rating emotional intensity on a scale from 1 (barely noticeable) to 10 (overwhelming).
2. **Management Framework:** Introduce the two-step process: **First Express, Then Regulate.**
3. **Emotion Regulation Steps (Expanded):**

- **Step 1: Mindful Experiencing:** Briefly observe and describe the emotion as it is in the body and mind (“I notice a tightness in my chest and a thought that I’m failing”), without trying to push it away or get swept away by it.
- **Step 2: Understanding the Source with Compassion:** Explore the possible roots of the emotion. **The goal is insight, not self-criticism.** Emphasize that all emotions serve a function (e.g., fear protects, anger signals a boundary crossed) and often have understandable origins, such as:
- **Learned Patterns from the Past:** A current feeling of being “not good enough” might be much stronger if you frequently received harsh criticism as a child.
- **Current Stressors & Thoughts:** As detailed in the expanded list below (Underlying Thoughts/Beliefs, Unmet Needs, Physical State).
- **Step 3: Finding a Healthy Outlet:** Channel the emotional energy adaptively. Examples include:
- **Creative Expression: Writing a letter (to a person, to the emotion itself, or an unsent farewell letter to a lost opportunity), drawing, or physical activity.**
- **Soothing/Comforting Activities: Taking a warm bath, listening to calming music, practicing paced breathing.**
- **Problem-Solving Action: If the source is a solvable problem, taking one small step to address it.**
- **Step 4: Re-engaging Mindfully:** Gently shift attention back to the present moment and the next valued action, using the regulated emotional state.

1. **Emotion Reference List (Example):**

| **Emotion** | **Common Prompting Event** | **Related Interpretations** | **Typical Body Sensations** |
| --- | --- | --- | --- |
| **Anxiety** | Facing uncertainty, perceived threat | “Something bad is going to happen.” “I can’t handle this.” | Muscle tension, rapid heartbeat, restlessness, stomachache |
| **Sadness** | Loss, disappointment, helplessness | “I am alone.” “This will never get better.” | Heaviness in chest/limbs, low energy, tearfulness, sighing |
| **Anger** | Goal blocked, right violated, hurt | “This is unfair.” “I’ve been disrespected.” | Feeling hot, clenched jaw/fists, increased energy, tension |
| **Guilt** | Having acted against one’s own values | “I did something wrong.” “I am responsible for the harm.” | Knot in stomach, pressure in chest, desire to hide/repair |

- **Homework Task:**

**Task 2.1:Identifying and Managing Our Emotions**

Participants complete a table for three different situations, practicing the full skill chain from identification to regulation, with a focus on understanding the source.

| **Situation** | **Your Feeling & Intensity (1-10)** | **Possible Source/Root** | **Your Expression/Regulation Action** |
| --- | --- | --- | --- |
| *e.g., Had a disagreement with a close friend, and they left upset.* | **Feeling:** Guilty, Anxious  **Intensity:** Guilt-7, Anxiety-6 | **Thought/Belief:** “A good friend wouldn’t have said that. I ruined the relationship.”  **Past Experience:** History of being blamed for conflicts in family.  **Need:** Need for connection and to be seen as a good person. | **Step1 (Mindful):** Noted the stomach knot and self-critical thoughts.  **Step2 (Understand):** Recognized the thought was extreme (“ruined”) and linked to old family patterns.  **Step3 (Outlet):** Wrote an unsent letter to my friend to express my regret and perspective, then did 5 minutes of deep breathing.  **Step4 (Re-engage):** Decided to text them tomorrow to apologize for my tone and ask to talk. |

### ****Module 3: Building Emotional Stability****

- **Objective:** Skills adapted from Dialectical Behavior Therapy (DBT) for emotion regulation and distress tolerance skills from DBT (Linehan, 2015).This module aims to help participants understand that emotional sensitivity and impulsivity are common challenges, particularly under stress or during episodes of depression/anxiety. It is designed to equip participants with two core, evidence-informed skills: (1) proactively building emotional resilience through the daily accumulation of positive experiences, and (2) effectively coping with acute emotional crises using an in-the-moment stabilization technique (the STOP skill).
- **Video Content Synopsis:**

1. **Review & Introduction:**  Recapped emotion identification from Module 2. Introduced the concept of emotional stability as a protective factor against the maintenance cycles of depression and anxiety. Normalized heightened emotional vulnerability during low mood states, framing it as a common experience rather than a personal flaw.
2. **Skill 1:** Accumulating Positive Experiences **(The "Emotional Battery" Model):**

- Rationale: Explained the bidirectional relationship between behavior and mood. Depression and anxiety often lead to withdrawal and reduced engagement in rewarding activities, which further depletes mood and energy ("emotional battery"). Proactively scheduling and engaging in positive activities serves as a direct, behavioral method to counteract anhedonia, low motivation, and rumination.
- Key Instructions: Participants were instructed to:
- Plan Micro-Activities: Schedule at least one small, manageable, pleasant or meaningful activity daily (e.g., savoring a beverage, brief contact with nature).
- Engage Mindfully: Practice being fully present during the activity, intentionally noting associated sensory details or subtle shifts in feeling.
- Problem-Solve Barriers: Use provided emotion-activity lists or past experiences to generate ideas if experiencing motivational deficits.

1. **Skill 2**: The STOP Skill (Emotional First-Aid):

- Rationale: Introduced as a crisis survival skill for moments of overwhelming affect (e.g., panic, intense sadness, anger) where impulsive reactions may be detrimental.
- Step-by-Step Protocol:
- S – Stop: Cease all action and automatic reaction immediately.
- T – Take a step back: Physically or mentally create distance. Initiate one slow, deep breath to engage the parasympathetic nervous system.
  - – Observe: Objectively notice internal experiences (bodily sensations, automatic thoughts as mental events) and external reality (immediate environment).
- P – Proceed mindfully: Ask, "What does this situation need?" or "What is the most effective action for my well-being right now?" before choosing an intentional next step.

1. Summary: Contrasted the two skills: Skill 1 for daily capacity-building and Skill 2 for acute crisis management. Emphasized regular practice for skill consolidation.

- **Homework Task:**
  Participants were assigned two structured practice tasks for this part following the module.

**Task 3.1: Daily Positive Experience Tracker**

Purpose: To facilitate behavioral activation and attentional shift towards rewarding stimuli.

| Date | Planned Pleasant/Micro- Activity | Actual Completion & Brief Reflection |
| --- | --- | --- |
| e.g., 2023-10-26 | Walk outside for 10 minutes | Walked, noticed sunlight. Felt slightly calmer. |

**Task 3.2: STOP Skill Practice Log**

Purpose: To enhance meta-awareness of emotional triggers and reinforce the application of the distress tolerance skill.

| Date Situation/Emotional Trigger | Action Taken (Did I use STOP?) | Outcome & Learning |
| --- | --- | --- |
| e.g., 2023-10-25 | Felt a surge of anxiety after a critical email. | Stopped before replying. Took 3 breaths. Observed my catastrophic thinking. Prevented an impulsive reply. Drafted response later with more clarity. Felt more in control. |

### ****Module 4: Behavioral Activation****

- **Objective:** This module is designed to teach participants the core principles and techniques of Behavioral Activation (BA), a key component of Cognitive Behavioral Therapy (CBT). The primary goal is to help individuals with mild-to-moderate depression and anxiety break the vicious cycle of low mood leading to inactivity, which in turn worsens mood. Participants will learn to use structured activity scheduling, task breakdown, and graded physical exercise to systematically increase engagement in rewarding and mastery-oriented activities, thereby improving mood, energy, and a sense of control.
- **Video Content Synopsis:**

1. **Review & Introduction:**  Recapped emotion identification from Module 3.

**Introduce The Activity-Mood Cycle**:

- Explained the bidirectional link between behavior and emotion. It described how stress, low mood, or anxiety can drain energy and motivation, leading to withdrawal from activities.
- **Illustrated the negative cycle**: Negative Emotion → Reduced Activity & Social Withdrawal → Loss of Pleasure/Mastery → Worsening Mood. Conversely, it introduced the recovery cycle facilitated by BA: Planned Action → Engagement in Activities → Increased Pleasure/Sense of Mastery → Improved Mood.
- Framed BA as a practical "entry point for change" to disrupt the downward spiral and rebuild a sense of agency.

1. **Core Strategy 1: Balanced Activity Scheduling (The "Four Quadrants"):**

- Participants were instructed to use a "Weekly Activity Schedule" to plan a balanced mix of four types of activities, aiming to restore routine and introduce positive experiences.
- **The Four Quadrants:**

1. **Social/Connection Activities:**Activities that foster a sense of being needed, understood, or valued (e.g., calling a supportive friend, spending time with loved ones). Rationale: Counters social withdrawal and loneliness common in depression/anxiety.
2. **Mastery/Control Activities:**Activities that provide a sense of accomplishment, competence, and independence (e.g., completing a task, learning something new, organizing a space). Rationale: Combats feelings of helplessness and low self-efficacy.
3. **Pleasure/Enjoyment Activities**: Activities pursued primarily for inherent enjoyment (e.g., hobbies, listening to music, playing with a pet). Rationale: Directly counteracts anhedonia (loss of pleasure) and introduces positive emotion.
4. **Self-Soothing/Sensory Activities:** Activities that engage the five senses to promote calm and present-moment awareness (e.g., taking a warm bath, mindful breathing, spending time in nature). Rationale: Provides tools for managing physiological arousal associated with anxiety and stress.
5. **Core Strategy 2: Task Breakdown (Graded Task Assignment):**

- Introduced as a method to overcome avoidance and feelings of being overwhelmed by large or complex tasks.
- Step-by-Step Protocol:

1. Deconstruct: Break a daunting task into a logical sequence of smaller, concrete steps.
2. Start Small: Begin with the first step that feels achievable, regardless of how small.
3. Acknowledge Effort: Consciously acknowledge completion of each step as a success.
4. Gradually Build: Slowly increase the number or difficulty of steps over time as confidence grows.

- Example Provided: The multi-step process of mowing a lawn was broken down across several days.

1. **Core Strategy 3: Graded Physical Exercise:**

- Presented exercise as a potent behavioral antidepressant and anxiolytic. Benefits were summarized succinctly, emphasizing both physiological (e.g., reduces stress hormones, improves sleep) and psychological effects (e.g., improves mood, reduces anxiety symptoms, boosts self-esteem).
- Practical Guidance for Getting Started (addressing low motivation):
- Schedule exercise as a fixed part of the routine.
- Start with very low-intensity activities on difficult days.
- Use social accountability (exercise with a friend/group).
- Use self-reward and practice self-compassion for missed days.
- Set realistic short- and long-term goals.

1. **Summary:**

Emphasized that consistent practice in scheduling a mix of activities, breaking down barriers, and incorporating movement can systematically rebuild a positive activity-mood cycle.

- **Homework Task:**
  Participants were assigned the following tasks following the module to apply the BA principles.

**Task 4.1: Weekly Activity Schedule & Monitoring**Purpose: To facilitate balanced activity planning and provide direct feedback on the relationship between activity engagement and mood.

| Date & Time | Planned Activity (Specify Quadrant: S/M/P/So) | Completed? (Y/N) | Pleasure Rating (0-100%) Before / After | Mastery Rating (0-100%) Before / After |
| --- | --- | --- | --- | --- |
| e.g., Mon 9 AM | M: Organize desk for 15 min | Y | 20% / 65% | 30% / 75% |
| e.g., Tue 7 PM | S: Call friend for 10 min | Y | 40% / 80% | 50% / 60% |
|  |  |  |  |  |
| *(Instructions: Try to schedule at least one activity from different quadrants across the week. The ratings are personal; there are no right or wrong scores.)* | | | |  |

**Task 4.2: Task Breakdown Practice**Purpose: To apply the skill of behavioral gradation to one avoided or procrastinated task.

- Task I've been avoiding: [e.g., "Preparing my tax documents"]
- Breakdown Steps:

1. [Gather all relevant paperwork in one box.]
2. [Download the necessary forms.]
3. [Fill out Section A for 30 minutes.]
4. [Review and complete Section B.]
5. [Double-check and file/submit.]

- My first achievable step is: [e.g., "Step 1: Gather paperwork by Friday."]

**Task 4.3: Weekly Exercise Log & Mood Tracking**Purpose: To monitor exercise frequency and its subjective impact on mood, reinforcing the behavior-mood link.

| Date | Type & Duration of Exercise | Mood Before (0-100, low-high) | Mood After (0-100, low-high) | Notes (e.g., easy/hard to start, how it felt) |
| --- | --- | --- | --- | --- |
| e.g., Wed | 20-min brisk walk | 45 | 70 | Felt sluggish at first, but more energized after. |
|  |  |  |  |  |
| *(Instructions: Any movement counts. Focus on consistency, not intensity.)* | | | | |

### ****Module 5: Structured Problem-Solving****

- **Objective:** This module teaches a simplified, core version of structured problem-solving, a key Cognitive Behavioral Therapy (CBT) skill. Designed for individuals experiencing low mood and anxiety, it aims to break the cycle of feeling overwhelmed and indecisive. The goal is to replace worry and inaction with a clear, 4-step thinking process, empowering participants to approach one manageable concern at a time.
- **Video Content Synopsis:**
- **Phase 1: Pause & Define (Instead of Reacting)**
- **Psychoeducation:**Acknowledges that stress and low mood can make problems seem huge and unsolvable. Introduces the idea of “hitting the mental pause button” before reacting.
- **Core Question:**“What is the one specific issue I want to tackle right now?” Encourages focusing on a single, current problem rather than everything at once.
- **Definition Tip:**Guide participants to define the problem as a **specific need or desired change**(e.g., “I need to feel less overwhelmed by my inbox” vs. “Work is stressful”).

**Phase 2: Brainstorm Options (No Judgment)**

- Instructs participants to quickly list 3-5 possible ways to address the defined problem.
- **Key Rule**: Emphasize that all ideas are valid in this phase. The goal is to generate possibilities, not critique them.
- **Prompt:** “What are some tiny, small, or medium steps I could take?”

**Phase 3: Pick a Path & Make a Plan**

- - Guides participants to review their brainstorm list and pick one option that seems most doable or promising right now.
  - **Simple Planning**: For the chosen option, plan the **very first step**. Make it concrete: **What** will I do? **When/Where** will I do it? (e.g., “I will spend 10 minutes tomorrow morning at my desk sorting emails into ‘urgent’ and ‘later’ folders.”).
  - **Decision Aid (Optional - Pros & Cons)**: Briefly introduces a simple “Pros and Cons” list for participants who feel stuck between two options, framing it as a tool to clarify thoughts.

**Phase 4: Try It & Reflect**

- Encourages participants to take the planned small step.
- **Reflective Learning**: Afterwards, guide them to ask: “What happened? What did I learn?” The focus is on learning from the attempt, not on achieving a perfect outcome. If it didn’t help, the lesson is valuable information for the next try.
- **Homework Task:**

**Task 5.1: My Problem-Solving Practice Sheet**

Purpose: To apply the 4-step process to one current, manageable concern in a low-effort, guided format.

| Step | Guiding Question | Your Brief Notes (Keep it simple) |
| --- | --- | --- |
| 1. Define | What’s one thing on my mind that I’d like to handle better? | e.g., “I’ve been putting off making a doctor’s appointment.” |
| 2.Brainstorm | What are 3 possible things I could do? (Any idea counts) | 1. Call the clinic tomorrow.  2. Ask my partner to remind me.  3. Look up online booking tonight. |
| 3. Pick & Plan | Which one small step will I try first? When/where? | “I will look up the clinic’s website tonight on my phone after dinner.” |
| 4. Reflect | After I tried it (or even if I didn’t): What did I notice or learn? | “I looked it up. It felt less scary once I saw the phone number. I learned that just getting information is a good first step.” |

### ****Module 6:**** ****Enhancing Social Connection and Communication****

- **Objective:** This module aims to address the social withdrawal and interpersonal difficulties commonly experienced during periods of depression and anxiety. Grounded in principles from Interpersonal Psychotherapy (IPT), it focuses on strengthening social support as a core protective factor. The objectives are: (1) to psychoeducate participants on the link between emotional well-being and secure interpersonal connections, (2) to teach basic, practical communication skills for expressing needs and feelings effectively, and (3) to provide a structured tool (the Life Event Timeline) to help individuals organize and share their personal experiences in a coherent way to foster understanding and support.
- **Video Content Synopsis:**

1. **Review & Introduction:**

Recapped Structured Problem-Solving from Module 5.

Introduce the Need for Connection in Times of Stress

- Explained how stress, low mood, and anxiety can trigger feelings of insecurity and isolation, often leading to social withdrawal.
- Framed the desire for connection as a fundamental human need rooted in attachment, and highlighted that actively seeking support is a proactive strategy for emotional recovery, not a sign of dependency.
- **Core Message**: Re-establishing or strengthening social bonds can directly counteract feelings of loneliness and helplessness, enhancing a sense of safety and belonging.

1. **Core Skill Set: Foundational Communication Skills**

Participants were introduced to three key communication principles to increase the likelihood of receiving understanding and support:

1. Choosing the Right Moment: Emphasized the importance of initiating conversations when both parties are likely to be receptive (considering timing, privacy, and emotional state).
2. Cultivating a Constructive Attitude: Encouraged an approach based on openness and kindness rather than blame or criticism. Stressed the importance of framing conversations around one’s own needs and feelings.
3. Practicing Effective Expression and Active Listening:

- **Expression (“I” Statements)**: Taught participants to express themselves using “I feel…” or “I think…” statements (e.g., “I’ve been feeling overwhelmed lately”) to describe their internal experience, rather than “You make me feel…” statements which can trigger defensiveness.
- **Listening**: Highlighted the value of giving the other person space to speak and making a genuine effort to understand their perspective without immediate judgment or interruption.

1. **Practical Tool: The Life Event Timeline**

Introduced as a narrative tool to help individuals organize complex feelings and experiences, making them easier to communicate.

**Instructions:** Participants are guided to draw a horizontal timeline representing a recent period (e.g., the past few months). They then mark significant events (both stressful and positive) as vertical lines along this timeline.

**Purpose**: This visual exercise helps users:

1. Objectively “see” the sequence and clustering of stressors.
2. Identify changes in their mood, behavior, or routines that occurred before, during, or after these events.
3. Clarify which contextual details are most important for a supportive other to know in order to understand their current emotional state.
4. Serve as a “story map” to reduce the feeling of being overwhelmed when trying to explain their situation to someone else.
5. **Homework Task:**

**Task 6.1: Simple Connection Practice**

Purpose: To gently practice reaching out for social support by having one brief, positive interaction with someone you trust.

Instructions:This days, your goal is to complete one small connection action. Follow these two simple steps:

**Step 1: Make a Tiny Plan**

- Choose ONE option from the list below:
- Send a supportive or friendly text message to a friend or family member (e.g., “Thinking of you, hope you’re having a good week.”).
- Have a short, low-pressure conversation (5-10 minutes) with someone in your household about a neutral or positive topic.
- Give a genuine compliment to someone (in person or online).

**Step 2: Quick Reflection**

After you complete your chosen action, take a moment to check in with yourself and mark below:

**After that interaction… (Check one)**

- I felt a bit more connected or understood. □
- My mood stayed about the same. □
- I felt a bit anxious or drained. □
- (Optional) A brief note on what I did: _____________________

### ****Module 7: Understanding Thoughts and Feelings****

- **Objective:** This brief introductory module aims to introduce the core Cognitive-Behavioral Therapy (CBT) concept of the interconnection between thoughts, feelings, and behaviors (the Cognitive Triangle). The primary goal is to help participants develop initial awareness of automatic negative thoughts (ANTs) that contribute to low mood and anxiety. The module does not aim for in-depth cognitive restructuring but focuses on the first critical skill: noticing and naming these thoughts in daily life
- **Video Content Synopsis:**

1. **The Thought-Feeling-Behavior Connection:**

Explained using a simple **triangle diagram**: Situations trigger automatic **Thoughts**, which instantly influence our **Feelings** and subsequently our **Actions/Behaviors.**

**Core Analogy:** “Thoughts are like the subtitles running in your mind. They tell you how to feel about what’s happening.”

1. **What Are Automatic Negative Thoughts (ANTs)?**

- Defined as the quick, fleeting thoughts that pop into our mind automatically, especially under stress. We often accept them as facts without question.
- Emphasized that while everyone has them, during periods of depression or anxiety, these thoughts tend to be disproportionately **negative, critical, or fearful**, fueling further distress.
- **Common Types (Simplified Examples):**
- **All-or-Nothing:** “I made a mistake, so I’m a total failure.”
- **Catastrophizing:** “If I feel anxious during the presentation, it will be a complete disaster.”
- **Mind Reading:** “They didn’t text back; they must be annoyed with me.”

1. **The First Step: Catch & Check the Thought**

- Introduced a simplified 2-step process for building awareness:
- **Catch It:** When you notice a sudden shift to a negative emotion (e.g., sadness, worry, irritation), pause and ask: **“What just went through my mind?”**
- **Check It:** Briefly note how much you believe that thought **in that moment** (0-100%). This simple act of labeling creates distance between the person and the thought.
- **Key Message:** “You don’t have to argue with the thought right away. Just noticing it is a powerful first step toward changing unhelpful patterns.”
- **Homework Task:**
- **Task 7.1: Thought-Spotting Practice**
  *Purpose: To build mindfulness of the link between situations, automatic thoughts, and feelings through simple observation.*

**Instructions:** Once a day, when you notice a dip in your mood, try to complete one row in the table below. You only need to fill in the three middle columns. The “Alternative Look” column is optional and can be skipped if you’re not ready

| \| **Situation (What happened?)** \| \| --- \| | **Feeling (What emotion?)** | **Automatic Thought (What went through my mind?)** | **Belief in Thought (0-100%)** | **Alternative Look (Optional: Is there another way to see it?)** |
| --- | --- | --- | --- | --- | --- |
| e.g., Saw a friend who seemed busy and didn’t stop to chat. | Sad, Rejected | “They’re ignoring me because I’m boring.” | 80% | “Maybe they were just in a hurry. They’ve been friendly before.” |

### ****Module 8: Mindful Moments for Daily Life****

- **Objective**: To introduce participants to three foundational mindfulness skills, each targeting a core aspect of experience in depression and anxiety: thoughts, behavior, and emotions. The module aims to teach practical “micro-practices” that can be applied in daily life to reduce reactivity and cultivate a calmer, more present-centered awareness.
- **Video Content Synopsis:**

1. **What is Mindfulness?**It was defined as intentionally paying gentle, non-judgmental attention to the present moment. The video emphasized its role not in eliminating negative feelings, but in changing one’s relationship with them.
2. **Part 1: The Thought (Managing the Inner Narrative)**

- **Key Idea:** “Thoughts are not facts.” We can learn to observe our thinking without getting swept away by it.
- **Micro-Practice – The Noticing Pause:** When a stressful or self-critical thought arises (e.g., “I can’t handle this”), simply pause and silently note: “I am having the thought that…”。 This creates a moment of distance between you and the thought.
- **Metaphor:** Thoughts are like passing clouds in the sky of your mind; you can notice them without having to hold onto them.

1. **Part 2: The Action (Breaking Autopilot)**

- **Key Idea:** We often live on “autopilot.” Mindful action brings us into the present moment through our senses.
- **Micro-Practice – The One-Minute Anchor:** Choose one routine activity (e.g., drinking water, walking to the door, washing hands). For one minute, fully focus on the physical sensations involved (e.g., the feel of the cup, the temperature of the water, the movement of your feet).
- **Goal:** To interrupt rumination or worry by grounding attention in the here-and-now.

1. **Part 3: The Emotion (Making Space for Feelings)**

- **Key Idea:** Emotions have physical components. By turning toward the body’s sensations with curiosity, we can process feelings without being overwhelmed by them.
- **Micro-Practice – The Body Check-In:** When you notice a strong emotion, pause for three breaths. Ask yourself: “Where do I feel this in my body?” (e.g., tightness in the chest, clenching in the jaw). Simply observe the sensation without trying to change it, breathing gently into that area.
- **Goal:** To foster acceptance of emotional experiences and reduce the struggle against them.
- **Homework Task:**

**Task 8.1: Mindful Moments Log**
*Purpose: To encourage brief, daily application of mindfulness skills to build familiarity and observe effects.*

| **Day** | **Which Micro-Practice Did I Try? (Thoughts/Actions/Emotions)** | **Brief Note (What I did & one thing I noticed)** |
| --- | --- | --- |
| e.g., Mon | Thoughts | Noticed the thought “This is too much.” Said to myself, “Having the ‘too much’ thought.” It felt less urgent. |
| e.g., Tue | Actions | Drank my coffee slowly for one minute, just tasting it. Felt a bit more grounded. |
| e.g., Wed | Emotions | Felt anxious before a call. Did a body check-in, felt tight shoulders. Just noticed it for 3 breaths. |
| *(Instructions: Try a different micro-practice each day, or repeat the one you find most helpful. The goal is not to “do it perfectly” but simply to practice noticing your experience in a new way.)* | | |
