# Supplementary material for: Efficacy of micro-video psychological training camp for reducing depression and anxiety and enhancing resilience: a randomized controlled trial
Source: BMC Psychiatry. 2026 Jan 23;26:173. doi: 10.1186/s12888-026-07807-6 (PMC12910843; doi:10.1186/s12888-026-07807-6)
Supplement: Supplementary file 2 — Supplementary Material 2 [file 12888_2026_7807_MOESM2_ESM.docx]

**Supplementary Material S3:** **All Outcome Measures in Chinese**

**一、人口学信息**

1. 序号
2. 称呼
3. 性别
4. 年龄
5. 文化程度
6. 职业
7. 是否独生子女
8. 童年时期的主要养育方式
9. 婚姻状况
10. 家庭成员
11. 吸烟
12. 饮酒
13. 三个月内是否经历紧张性生活事件（有，请注明）
14. 半年内是否接受过系统的心理咨询或心理治疗
15. 重大躯体疾病史
16. 精神疾病
17. 精神疾病遗传史
18. 目前是否正在服用精神科药物（何时服用-何时停止[若停止]，药物名称及计量）
19. 目前是否正在服用安眠药物（何时服用-何时停止[若停止]，药物名称及计量
20. 手机号码

**二、量表（下划线反向计分）**

**焦虑自评量表the Self-Rating Anxiety Scale (SAS)**

**指导语：以下列出了二十个条目，请您仔细阅读每一个条目，然后根据最近一周的实际情况和感受，判断每个陈述在多大程度上与您的状况相符**

***1=没有或很少；2=小部分时间；3=相当多时间；4=绝大部分或全部时间***

1. 我觉得比平常容易紧张和着急
2. 我无缘无故地感到害怕
3. 我容易心理烦乱或觉得惊恐
4. 我觉得我好像要发疯
5. 我觉得一切都很好，也不会发生什么不幸
6. 我手脚发抖打颤
7. 我因为头疼颈痛和背痛而苦恼
8. 我感觉容易衰弱和疲乏
9. 我觉得心平气和，并且容易安静坐着
10. 我觉得心跳得很快
11. 我因为一阵阵头晕而苦恼
12. 我有晕倒发作，或觉得要晕倒似的
13. 我吸气呼气都感到很容易
14. 我的手脚麻木和刺痛
15. 我因为胃痛和消化不良而苦恼
16. 我很短时间就要小便
17. 我的手脚常常是干燥温暖的
18. 我脸红发热
19. 我容易入睡并且一夜睡得很好
20. 我做噩梦

**抑郁自评量表the Self-Rating Depression Scale (SDS)**

**指导语：以下列出了二十个条目，请您仔细阅读每一个条目，然后根据最近一周的实际情况和感受，判断每个陈述在多大程度上与您的状况相符。**

***1=没有或很少；2=小部分时间；3=相当多时间；4=绝大部分或全部时间***

1. 我感到闷闷不乐，情绪低沉
2. 我觉得一天中早晨最好
3. 我一阵阵哭出来或觉得想哭
4. 我晚上睡眠不好
5. 我吃得与平常一样多
6. 我与异性密切接触时和以往一样感到愉快
7. 我发觉我的体重在下降
8. 我有便秘的苦恼
9. 我心跳比平常快
10. 我无缘无故地感到疲乏
11. 我的头脑和平常一样清楚
12. 我觉得经常做的事情并没有困难
13. 我觉得不安而平静不下来
14. 我对未来抱有希望
15. 我比平常容易生气激动
16. 我觉得做出决定是容易的
17. 我觉得自己是个有用的人，有人需要我
18. 我的生活过得很有意思
19. 我认为如果我死了，别人会生活得好些
20. 平常感兴趣的事我仍然感兴趣

**失眠严重程度量表The Insomnia Severity Index (ISI)**

**指导语：请您仔细阅读以下 7个问题，每个问题都描述了失眠可能带来的不同困扰。请根据最近两周的实际情况，选择最符合您个人感受的选项**

入睡困难：*1=无；2=轻度；3=中度；4=重度；5=非常严重*

无法维持较长的睡眠：*1=无；2=轻度；3=中度；4=重度；5=非常严重*

太早醒：*1=无；2=轻度；3=中度；4=重度；5=非常严重*

您满意自己最近的睡眠状态吗？：*1=非常不满意；2=不满意；3=中等；4=满意；5=非常满意*

睡眠问题是否有干扰到您的日常生活功能？（例如白天疲劳工作表现/日常琐事专注力记忆力情绪等）。：*1=完全无干扰；2=一点；3=稍微；4=很多；5=非常多*

他人是否有注意到您的生活品质因睡眠问题受到影响？：*1=完全没注意；2=一点；3=稍微；4=很多；5=非常注意*

最近的睡眠问题是否令您担心/困扰？：*1=完全无担心；2=一点；3=稍微；4=很多；5=非常担心*

**心理韧性量表The Connor-Davidson Resilience Scale (CD-RISC)**

**指导语：请根据过去一个月的经验，选择你对以下各陈述句子的同意程度。如果有些特殊情境并未发生，则回答假如真的发生了，你的感受会是怎样。**

**评分：*1=完全不符合；2=比较不符合；3=说不清；4=比较符合；5=完全符合***

1.不管环境发生什么变化，我都能够适应

2.当面对压力时，至少有一个亲近而且可靠的人可以帮助我

3.当问题无法清楚地获得解决时，我有时相信命运或神能帮助我

4.不管我的人生路途发生什么事情，我都能处理

5.过去的成功让我有信心去迎接新的挑战和困难

6.当面对问题时，我会试着去看事情好的一面

7.我相信经历过磨炼后，我会变得更坚强

8.在生病受伤或苦难之后，我很容易就能调整过来

9.不管事情好坏，我相信事出必有因

10.不管结果如何，我都会尽最大的努力

11.即使有困难，我也相信能够实现自己的目标

12.即使情况看起来没有希望，我仍然不放弃

13.当压力或危机来到时，我知道在哪里可以获得帮助

14.就算在压力下，我仍然能够精神集中地思考问题

15.我宁愿自力更生解决问题，而不会听任别人摆布

16.我不会轻易地被失败打倒

17.当处理生活中的挑战和苦难时，我想我是一个坚强的人

18.如果有必要，我会做出不受欢迎或困难的决定

19.我能够处理一些不愉快或痛苦的感受，例如悲伤害怕和生气

20.在处理生活难题时，有时我会按第一感觉办事

21.在我的生活中，我有明确的目标

22.我觉得可以控制自己的生活

23.我喜欢挑战

24.不管在人生路途上遇到什么障碍，我都会努力达到我的目标

25.我为自己的成就而感到自豪

**感知社会支持量表The Perceived Social Support Scale (PSSS)**

**指导语：请您根据自己的实际情况在每句后面选择一个最符合您情况的选项。**

***1=极不同意；2=比较不同意；3=有点不同意；4=不确定；5=有点同意；6=比较同意；7=完全同意***

1.在我遇到问题时有些人（领导亲戚同事）会出现在我的身旁

2.我能够与有些人（领导亲戚同事）共享快乐与忧伤

3.我的家庭能够切实具体地给我帮助

4.在需要时我能够从家庭获得感情上的帮助和支持

5.在需要时有些人（领导亲戚同事）是安慰我的真正源泉

6.我的朋友们能真正的帮助我

7.在发生困难时我可以依靠我的朋友们

8.我能与自己的家庭谈论我的难题

9.我的朋友们能与我分享快乐与忧伤

10.在我的生活中有些人（领导亲戚同事）关心着我的感情

11.我的家庭能心甘情愿协助我作出各种决定

12.我能与朋友们讨论自己的难题

**应对方式量表The Simple** **Coping Style Questionnaire (SCSQ)**

**指导语：以下列出的是当您在生活中遭受挫折或打击，或者遇到困难时可能采用的态度或做法。请您仔细阅读每一项，然后选择一项符合您做法的选项。**

***1=不采用；2=偶尔采用；3=有时采用；4=经常采用***

1.通过工作学习或其他一些活动解脱

2.与人交谈，倾诉内心的烦恼

3.尽量看到事物好的一面

4.改变自己的想法，重新发现生活中什么重要

5.不把问题看得太严重

6.坚持自己的立场，为自己想得到的斗争

7.找出几种不同的解决问题的方法

8.向亲戚朋友或同学寻求建议

9.改变原来的一些做法或自己的一些问题

10.借鉴他人处理类似困难情景的办法

11.寻求业余爱好，积极参加文体活动

12.尽量克制自己的失望悔恨悲伤和愤怒感情

13.试图休息或休假，暂时把问题（烦恼）抛开

14.通过吸烟喝酒服药和吃东西来消除烦恼

15.认为时间会改变现状，唯一要做的便是等待

16.试图忘记整个事情

17.依靠别人解决问题

18.接受现实，因为没有其它办法

19.幻想可能会发生某种奇迹改变现状

20.自己安慰自己

**三、满意度调查。*1=非常不赞同；2=不赞同；3=一般；4=赞同***

1. 小程序的操作简单易懂。
2. 自助式微视频治疗的形式我可以接受。
3. 我觉得在观看完微视频之后有必要进行作业的练习。
4. 微视频和作业的内容容易理解。
5. 微视频治疗对我来说是有帮助的。
6. 我更愿意有人当面教授我这些技能，而不是我一个人在手机上练习。
7. 相比面对面心理治疗，我喜欢这样的方式。
