# Supplementary material for: Efficacy of micro-video psychological training camp for reducing depression and anxiety and enhancing resilience: a randomized controlled trial
Source: BMC Psychiatry. 2026 Jan 23;26:173. doi: 10.1186/s12888-026-07807-6 (PMC12910843; doi:10.1186/s12888-026-07807-6)
Supplement: Supplementary file 3 — Supplementary Material 3 [file 12888_2026_7807_MOESM3_ESM.docx]

**Supplementary Material S7: Safety Data and Adverse Events Reporting**

1. **Definitions and Monitoring Methods**

In accordance with Chinese clinical trial regulations and ethical requirements, this study defined and implemented the following procedures for Adverse Events (AEs) and Serious Adverse Events (SAEs):

- **Adverse Event (AE)**: Any unfavorable or unintended medical occurrence in a participant during the study period, which did not necessarily have a causal relationship with the study intervention (MVPTC).
- **Serious Adverse Event (SAE)**: Any AE that resulted in death, was life-threatening, required inpatient hospitalization or prolongation of existing hospitalization, resulted in persistent or significant disability/incapacity, or was a significant medical event. For this study, **newly emergent active suicidal ideation or behavior** was pre-specified as an SAE.
- **Severity Grading:**
- **Mild**: Tolerable, does not interfere with daily function, and requires no special intervention.
- **Moderate**: Causes discomfort sufficient to interfere with daily function or recovery, and may require clinical attention or intervention.
- **Severe**: Life-threatening, causes significant incapacity, and requires immediate emergency medical intervention.

**Safety Monitoring and Data Collection Pathways:**

Given the self-guided, digital nature of the intervention, the study did not incorporate a prospective, systematic electronic AE questionnaire. Safety data collection relied on two pre-defined, passive monitoring channels:

1. **Risk-Alert-Triggered Proactive Follow-up**: Participant responses during scheduled online assessments (T1-T4) were monitored by automated algorithms for predefined risk indicators (e.g., specific scores on suicidality items). If triggered, **a blinded research assistant** initiated a standardized safety protocol within 24 hours, involving telephone contact for clinical risk assessment and documentation. This served as the primary mechanism for identifying potential SAEs and significant clinical deterioration.

2. **Participant-Initiated Reports**: The contact information for the research coordinator ("assistant") was provided in the informed consent documents. Participants were instructed to proactively report any significant distress or adverse reactions experienced during the intervention via this channel. All such communications were logged and archived.

1. **Recording, Assessment, and Management Procedures**

All potential AE/SAE information gathered through the above channels was managed as follows:

1. **Recording**: Details including time of onset, clinical manifestations, actions taken, duration, outcome, and preliminary assessment of relationship to the intervention were documented in the study Case Report Form (CRF) or a dedicated safety event log.
2. **Assessment and Adjudication**: Upon study completion, a retrospective review of all safety records was conducted independently by two clinical experts (attending psychiatrists or above) blinded to group allocation. They jointly adjudicated whether incidents met AE/SAE criteria, assessed severity, and determined causality in relation to the MVPTC intervention using a five-point scale: **definitely related, probably related, possibly related, unlikely related, unrelated**. Discrepancies were resolved through consultation with a third senior expert.
3. **Management and Reporting:**
   - All AEs were followed until resolution or stabilization.
   - Any SAE would have been reported to the Principal Investigator, the Ethics Committee, and relevant regulatory authorities within **24 hours** of awareness, with subsequent management per the Ethics Committee's guidance.
   - As per the approved ethical protocol, participants would have received necessary medical care and appropriate compensation for any trial-related injury.
4. **Summary of Adverse Events**

The independent experts' retrospective review of all safety monitoring records identified the following adjudicated event during the study period (from post-randomization through the 3-month follow-up).

**Table. Summary of Adverse Events Reported During the Study.**

| **Description of Adverse Event** | **MVPTC Group (n=97)** | **Waitlist Control Group (n=107)** | **Severity** | **Causality Assessment** |
| --- | --- | --- | --- | --- |
| **Worsening of depressive symptoms, leading to seeking outpatient psychiatric care and initiation of regular medication.** | 1 | 0 | Moderate | Possibly Related |
| **Total Number of Unique AEs** | 1 | 0 |  |  |

**Notes:**

1. This event was adjudicated as a **non-serious adverse event (AE)** of moderate severity. The participant in the intervention group contacted the assistant after completing Module 4, reporting increased low mood. Following assessment by the assistant, the participant was advised and assisted in seeking outpatient psychiatric consultation, where medication was recommended. According to the study protocol, this participant was classified as a **"treatment changer"**; their data was excluded from the primary efficacy (ITT) analysis. However, the participant completed all intervention modules, the post-test (T2), and the 1-month follow-up (T3) assessment. The event resolved following the initiated medical intervention.

2. **No Serious Adverse Events (SAEs),** including suicidal attempts, suicidal behavior, hospitalization, or death, were reported or identified through either monitoring channel in either group.

3. No other AEs were reported or recorded.

1. **Conclusion**

Based on the standardized safety monitoring and assessment procedures implemented in this study (risk-alert-triggered follow-up and participant-initiated reports, followed by retrospective expert review), the self-guided digital psychological intervention "Micro-Video Psychological Training Camp" (MVPTC) demonstrated a favorable safety and tolerability profile in adults with mild-to-moderate depressive or anxiety symptoms. Only one non-serious, possibly related AE of moderate severity was reported during the entire study period. **No serious adverse events related to the intervention were observed.**
