# Supplementary material for: Efficacy of micro-video psychological training camp for reducing depression and anxiety and enhancing resilience: a randomized controlled trial
Source: BMC Psychiatry. 2026 Jan 23;26:173. doi: 10.1186/s12888-026-07807-6 (PMC12910843; doi:10.1186/s12888-026-07807-6)
Supplement: Supplementary file 4 — Supplementary Material 4 [file 12888_2026_7807_MOESM4_ESM.docx]

****Table S1. Participant completion and dropout by group and assessment time point.****

| Group | Randomized (n) | T2 (Post-intervention) | T3 (1-month follow-up) | T4 (3-month follow-up) |
| --- | --- | --- | --- | --- |
| Intervention Group | 97 | 62 (63.9%) | 53 (54.6%) | 46 (47.4%) |
| Control Group | 107 | 98 (91.6%) | 84 (78.5%) | 68 (63.6%) |
| ****Total**** | ****204**** | ****160 (78.4%)**** | ****137 (67.2%)**** | ****114 (55.9%)**** |

Note: Percentages indicate the proportion of participants remaining relative to the number randomized within each group.

****Table S2. Model Fit Statistics for Linear Mixed Models****

| Outcome | Structure | AIC | BIC | Log-Likelihood | ΔAIC |
| --- | --- | --- | --- | --- | --- |
| ****SDS**** | Unstructured | 4183.18 | 4231.62 | -2080.59 | 0.00 |
|  | Compound Symmetry | 4185.18 | 4237.84 | -2080.59 | 2.00 |
| ****SAS**** | Unstructured | 4029.31 | 4077.75 | -2003.65 | 0.00 |
|  | Compound Symmetry | 4031.31 | 4083.97 | -2003.65 | 2.00 |
| ****CD-RISC**** | Unstructured | 773.20 | 820.86 | -375.60 | 0.00 |
|  | Compound Symmetry | 775.20 | 827.00 | -375.60 | 2.00 |

Note: Lower AIC and BIC values indicate better model fit. The unstructured covariance structure was selected for all final analyses based on superior fit statistics.

# **Table S3: Sensitivity Analyses**

# ****A. Complete-case Analysis****

Analysis of participants with data at all four assessment time points.

| Outcome | T1 (n) | T2 (n) | T3 (n) | T4 (n) | Complete 4-timepoints (n) | Completion Rate (%) |
| --- | --- | --- | --- | --- | --- | --- |
| SDS | 204 | 156 | 131 | 113 | 104 | 51.0 |
| SAS | 204 | 156 | 131 | 113 | 104 | 51.0 |

Note: Complete cases defined as participants with data at all four assessment time points (baseline, post-intervention, 1-month, and 3-month follow-ups).

## ****B. Clinical Significance at 3-Month Follow-up****

Remission defined as SDS score <53 and SAS score <50.

| Outcome | Group | Timepoint | N | Mean (SD) | Remission n (%) |
| --- | --- | --- | --- | --- | --- |
| ****SDS**** | Intervention | T4 | 46 | 47.50 (10.71) | 31 (67.4%) |
|  | Control | T4 | 68 | 52.24 (12.52) | 34 (50.0%) |
| ****SAS**** | Intervention | T4 | 46 | 43.53 (10.43) | 35 (76.1%) |
|  | Control | T4 | 68 | 46.77 (10.43) | 40 (58.8%) |

Note: Clinical cut-offs: SDS <53 indicates no clinically significant depression; SAS <50 indicates no clinically significant anxiety.

## ****C. Covariance Structure Comparison for Linear Mixed Models****

Lower AIC and BIC values indicate better model fit.

| Outcome | Covariance Structure | AIC | BIC | ΔAIC |
| --- | --- | --- | --- | --- |
| ****SDS**** | Unstructured | 4183.18 | 4231.62 | 0.00 |
|  | Compound Symmetry | 4185.18 | 4237.84 | 2.00 |
| ****SAS**** | Unstructured | 4029.31 | 4077.75 | 0.00 |
|  | Compound Symmetry | 4031.31 | 4083.97 | 2.00 |
| ****CD-RISC**** | Unstructured | 773.20 | 820.86 | 0.00 |
|  | Compound Symmetry | 775.20 | 827.00 | 2.00 |

Note: The unstructured covariance structure was selected for all final analyses based on superior fit statistics (lower AIC and BIC values).

## ****D. Outlier Analysis****

Outliers defined as values >3 median absolute deviations (MAD) from the median.

| Variable | Timepoint | N | Median | MAD | Outliers n (%) |
| --- | --- | --- | --- | --- | --- |
| SDS1 | T1 | 204 | 61.25 | 6.49 | 0 (0.00%) |
| SDS2 | T2 | 156 | 55.00 | 9.27 | 0 (0.00%) |
| SDS3 | T3 | 131 | 53.75 | 11.12 | 0 (0.00%) |
| SDS4 | T4 | 113 | 51.25 | 12.97 | 0 (0.00%) |
| SAS1 | T1 | 204 | 50.00 | 7.41 | 0 (0.00%) |
| SAS2 | T2 | 156 | 45.00 | 7.41 | 2 (1.28%) |
| SAS3 | T3 | 131 | 43.75 | 9.27 | 1 (0.76%) |
| SAS4 | T4 | 113 | 43.75 | 9.27 | 1 (0.88%) |

Note: Interpretation: <5% = Acceptable, ≥5% = High. All variables showed acceptable levels of outliers.


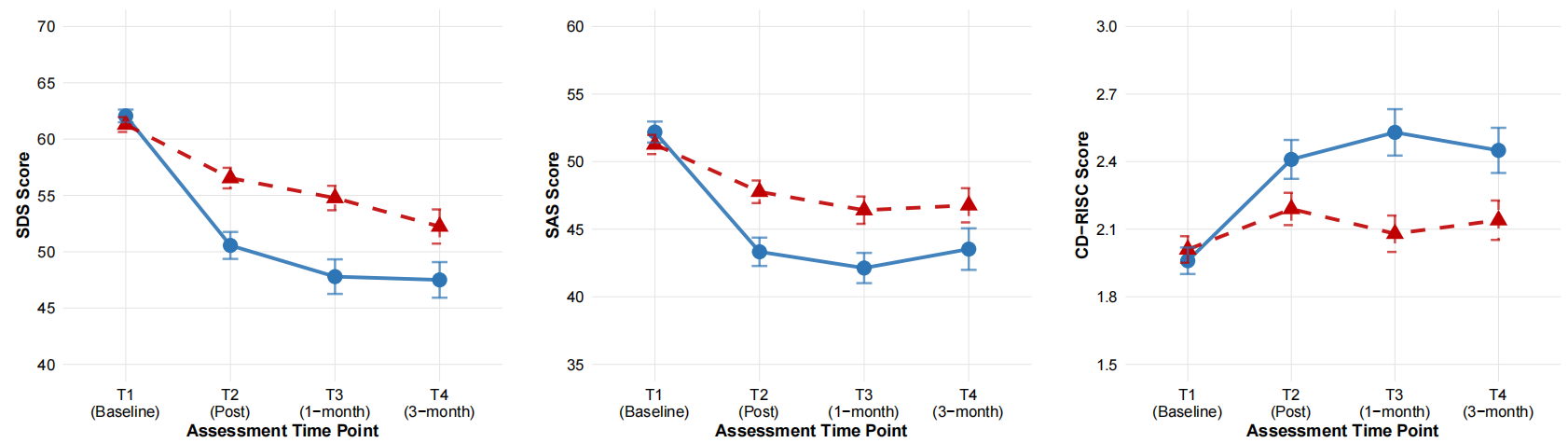


Figure S1. Trajectories of primary outcomes across four assessment time points. (a) Depressive symptoms measured by SDS. (b) Anxiety symptoms measured by SAS. (c) Psychological resilience measured by CD-RISC. Data points represent observed means with error bars indicating standard errors. Solid blue line with circles = Micro-Video Psychological Training Camp intervention group; dashed red line with triangles = waitlist control group. T1 = baseline, T2 = post-intervention (4 weeks), T3 = 1-month follow-up, T4 = 3-month follow-up. Sample sizes for intervention group: n=97 (T1), 62 (T2), 53 (T3), 46 (T4); control group: n=107 (T1), 98 (T2), 84 (T3), 68 (T4).
